# Impact of Prasugrel and Ticagrelor on Platelet Reactivity in Patients With Acute Coronary Syndrome: A Meta-Analysis

**DOI:** 10.3389/fcvm.2022.905607

**Published:** 2022-06-09

**Authors:** Lulu Dai, Jiawei Xu, Yuerong Jiang, Keji Chen

**Affiliations:** ^1^National Clinical Research Center for Chinese Medicine Cardiology, Xiyuan Hospital, China Academy of Chinese Medical Sciences, Beijing, China; ^2^Graduate School of Beijing University of Chinese Medicine, Beijing, China

**Keywords:** prasugrel, ticagrelor, platelet reactivity, acute coronary syndrome, meta-analysis

## Abstract

**Background:**

This meta-analysis mainly aimed to compare the impact of prasugrel and ticagrelor on platelet reactivity (PR) in patients with acute coronary syndrome (ACS).

**Methods:**

We searched four electronic databases to identify randomized controlled trials and cohort studies comparing the impact of prasugrel and ticagrelor on PR in patients with ACS. We performed group analyses according to three detection methods, drug dose [loading dose (LD) and maintenance dose (MTD)] and LD effect time, and assessed the robustness of the results through sensitivity analysis.

**Results:**

Twenty-five studies with 5,098 patients were eligible. After LD, the incidence of high on-treatment platelet reactivity (HTPR) of ticagrelor was significantly lower than that of prasugrel within 6–18 h based on vasodilator-stimulated phosphoprotein (VASP) test [RR = 0.25 (0.07, 0.85), *P* = 0.03], there was no significant difference between ticagrelor and prasugrel in the following results: platelets inhibitory effect within 24–48 h based on VerifyNow P2Y12 (VN) assay (*P* = 0.11) and VASP test (*P* = 0.20), and the incidence of HTPR within 2–6 h based on VN assay (*P* = 0.57) and within 24–48 h based on VN assay (*P* = 0.46) and VASP test (*P* = 0.72), the incidence of low on-treatment platelet reactivity (LTPR) within 6–18 h based on VASP test (*P* = 0.46) and 48 h based on VN assay (*P* = 0.97) and VASP test (*P* = 0.73). After MTD, the platelet inhibitory effect of ticagrelor was stronger than that of prasugrel based on VN assay [WMD = −41.64 (−47.16, −36.11), *P* < 0.00001]and VASP test [WMD = −9.10 (−13.88, −4.32), *P* = 0.0002], the incidence of HTPR of ticagrelor was significantly lower than that of prasugrel based on VN assay [RR = 0.05 (0.02, 0.16), *P* < 0.00001], the incidence of LTPR of ticagrelor was significantly higher than prasugrel based on VN assay [RR = 6.54 (4.21, 10.14), *P* < 0.00001] and VASP test [RR = 2.65 (1.78, 3.96), *P* < 0.00001], the results of Multiple Electrode Aggregometry (MEA) test was inconsistent with the other two detection methods in platelet inhibitory effect and the incidence of HTPR and LTPR. There was no significant difference between ticagrelor and prasugrel in the following clinical outcomes: all-cause death (*P* = 0.86), cardiovascular death (*P* = 0.49), myocardial infarction (*P* = 0.67), stroke (*P* = 0.51), target vessel revascularization (*P* = 0.51), stent thrombosis (*P* = 0.90), TIMI major bleeding (*P* = 0.86) and bleeding BARC type ≥ 2 (*P* = 0.77). The risk of bleeding BARC type 1 of ticagrelor was significantly higher than prasugrel [RR = 1.44 (1.03, 2.02), *P* = 0.03].

**Conclusions:**

Compared with prasugrel, ticagrelor might have a stronger platelet inhibition effect, with a lower incidence of HTPR and a higher incidence of LTPR and bleeding BARC type 1, while there might be no significant difference in the risk of thrombosis/ischemic, bleeding BARC Type ≥ 2 and TIMI major bleeding. A higher incidence of LTPR might indicate a higher risk of bleeding BARC type 1. The results of VN assay were consistent with that of VASP test, and not with the MEA test.

**Systematic Review Registration:**

https://www.crd.york.ac.uk/prospero/display_record.php?ID=CRD42022304205, identifier: CRD42022304205.

## Introduction

Platelets are a key part of the occurrence and development of adverse atherosclerotic thrombotic events. Inhibition of platelet function reduces the incidence of cardiovascular adverse events (1). Dual antiplatelet therapy (DAPT), which consists of aspirin and a P2Y12 inhibitor, is currently standard of care for secondary prevention oral antithrombotic therapy in patients with acute coronary syndrome (ACS) and post-percutaneous coronary intervention (2–4). Clopidogrel, a second-generation thienopyridine, is currently most widely used P2Y12 inhibitor in clinical practice (5). However, the pharmacological limitations of clopidogrel mainly include weak platelet inhibition, slow-onset and long duration of action, and significant pharmacodynamic and pharmacokinetic variability among individuals (6). Studies have shown that individuals treated with clopidogrel exhibit a wide range of response variability due to both genetic and non-genetic factors, such as genetic polymorphism, low bioavailability and drug interactions (7, 8). High on-clopidogrel platelet reactivity during DAPT is an important sign of vascular risk, especially stent thrombosis, in patients with ACS (9).

Therefore, new P2Y12 inhibitors, such as ticagrelor and prasugrel, were developed to overcome major pharmacokinetic limitations of clopidogrel. Compared with clopidogrel, ticagrelor and prasugrel are more potent P2Y12 inhibitors, which can produce a more reliable and stronger platelet inhibitory effect and have also shown superiority in the reduction of ischemic vascular events, however, increase the incidence of bleeding events (10–13). Ticagrelor, a cyclopenthyl-triazolopyrimidine, is a reversible antagonist of platelet P2Y12 receptor and does not need to be converted to active metabolites, while prasugrel, a third-generation thiophene pyridine, converts into its active metabolite *in vivo* and then irreversibly inhibits the p2y12 receptor (6). The antiplatelet mechanism of action of ticagrelor and prasugrel is different, and the responsiveness to platelets is also different, which possibly result in differences in biological and clinical outcomes. Platelet reactivity (PR), highly variable and associated with thrombosis and bleeding events, is a critical pharmacodynamic ingredient in patients receiving antiplatelet therapy (14). Evidence supports the association of high on-treatment platelet reactivity (HTPR) and low on-treatment platelet reactivity (LTPR) with ischemic events and bleeding events, respectively (9, 15).

There have been many meta-analyses comparing adverse clinical outcomes between prasugrel and ticagrelor, such as cardiovascular death, stroke, myocardial infarction and bleeding events, while relatively fewer meta-analyses directly comparing the impact of the two drugs on PR. In the latest meta-analysis comparing the impact of two drugs on PR, only two of the included studies reported HTPR based on VerifyNow P2Y12 (VN) assay, and none reported LTPR based on VN assay (16). In addition, the latest meta-analysis didn't assess robustness of results with high heterogeneity by sensitivity analyses. Currently, few meta-analyses compare the incidence of LTPR in the treatment of prasugrel and ticagrelor. Therefore, we conducted a meta-analysis with a more comprehensive search of literatures, and included randomized controlled trials (RCTs) and cohort studies that comparing the impact of ticagrelor and prasugrel of loading dose (LD) and maintenance dose (MTD) on PR according to VN assay, vasodilator-stimulated phosphoprotein (VASP) test and Multiple Electrode Aggregometry (MEA), further summarized the risk ratios of HTPR and LTPR of the two drugs, to compare the pharmacodynamic differences of that, while clinical outcomes in studies that met the eligibility criteria were also compared.

## Methods

Our meta-analysis was performed based on the Preferred Reporting Items for Systematic Reviews and Meta-Analyses (PRISMA) and the Meta-analysis of Observational Studies in Epidemiology (MOOSE) reporting guidelines (17, 18). Protocol of this meta-analysis was registered in the International Prospective Register of Systematic Reviews (PROSPERO), registration number: CRD42022304205.

### Literature Search

A comprehensive search of PubMed, Embase, Web of Science and Cochrane library was conducted without language restrictions through a combination of Boolean logical operators with keywords, which including “Prasugrel Hydrochloride”, “Hydrochloride, Prasugrel”, “Prasugrel HCl”, “HCl, Prasugrel”, “CS 747”, “747, CS”, “CS-747”, “CS747”, “Prasugrel”, “Efient”, “Effient”, “LY 640315”, “640315, LY”, “LY640315”, “LY-640315”, “Ticagrelor ”, “Brilique”, “AZD 6140”, “AZD6140”, “AZD-6140”, “Brilinta”, “platelet reactivity”, “vasodilator-stimulated phosphoprotein”, “vasodilator stimulated phosphoprotein”, “VerifyNow”, “Verify Now”, “multiple electrode aggregometry ”, “Acute Coronary Syndrome”, “Acute Coronary Syndromes”, “Coronary Syndrome, Acute”, “Coronary Syndromes, Acute”, “Syndrome, Acute Coronary”, “Syndromes, Acute Coronary”, “st segment elevation acute myocardial infarction”, “non-st segment elevation acute myocardial infarction” and “unstable angina” (inception to Apr 26, 2022). We also examined the references of relevant meta-analyses and reviews to track potentially relevant literatures. Table 1 shows the search strategy of PubMed. The search strategies of Embase, Web of science, and Cochrane are detailed in Supplementary Tables 1–3 respectively.

**Table 1:** PubMed search strategy.

**Search**	**Query**
#1	Search: (“Prasugrel Hydrochloride”[Mesh]) OR (Prasugrel Hydrochloride[Title/Abstract]) OR (Hydrochloride, Prasugrel[Title/Abstract]) OR (Prasugrel HCl[Title/Abstract]) OR (HCl, Prasugrel[Title/Abstract]) OR (CS 747[Title/Abstract]) OR (747, CS[Title/Abstract]) OR (CS-747[Title/Abstract]) OR (CS747[Title/Abstract]) OR (Prasugrel[Title/Abstract]) OR (Efient[Title/Abstract]) OR (Effient[Title/Abstract]) OR (LY 640315[Title/Abstract]) OR (640315, LY[Title/Abstract]) OR (LY640315[Title/Abstract]) OR (LY-640315[Title/Abstract])
#2	Search: (“Ticagrelor”[Mesh]) OR (Ticagrelor[Title/Abstract]) OR (Brilique[Title/Abstract]) OR (AZD 6140[Title/Abstract]) OR (AZD6140[Title/Abstract]) OR (AZD-6140[Title/Abstract]) OR (Brilinta[Title/Abstract])
#3	Search: (platelet reactivity[Title/Abstract])
#4	Search: (vasodilator-stimulated phosphoprotein[Title/Abstract]) OR (vasodilator stimulated phosphoprotein[Title/Abstract]) OR (VerifyNow[Title/Abstract]) OR (Verify Now[Title/Abstract]) OR (multiple electrode aggregometry [Title/Abstract])
#5	Search: (“Acute Coronary Syndrome”[Mesh]) OR (Acute Coronary Syndrome[Title/Abstract]) OR (Acute Coronary Syndromes[Title/Abstract]) OR (Coronary Syndrome, Acute[Title/Abstract]) OR (Coronary Syndromes, Acute[Title/Abstract]) OR (Syndrome, Acute Coronary[Title/Abstract]) OR (Syndromes, Acute Coronary[Title/Abstract]) OR (st segment elevation acute myocardial infarction[Title/Abstract]) OR (non-st segment elevation acute myocardial infarction[Title/Abstract]) OR (unstable angina[Title/Abstract])
#6	Search: #1 AND #2 AND #3 AND #4 AND #5

### Eligibility Criteria

Inclusion criteria: (1) RCTs or cohort studies; (2) comparison of prasugrel and ticagrelor on PR; (3) patients with ACS took aspirin combined with standard doses of prasugrel [180 mg (LD), 90 mg bid (MTD)] or ticagrelor [60 mg (LD), 10 mg bid (MTD)] orally; (4) studies reported one or more of the five outcome measures: P2Y12 response unit (PRU), platelet response index (PRI), the area under the curve of aggregation tracing (AUC), HTPR and LTPR.

Exclusion criteria: (1) duplicate publications; (2) conference abstracts or no full text; (3) no outcome measures of interest; (4) non-standard dosage of ticagrelor or prasugrel; (5) required data was not available.

### Literature Selection

NoteExpress (version 3.2) was applied to manage retrieved records. Two researchers reviewed titles and abstracts independently of each other based on inclusion and exclusion criteria. Potential literatures that met the criteria required further full-text review. If there were any disagreements on the list of eligible literatures, the list would be reviewed by a third researcher as an arbitrator.

### Data Extraction

After pilot extraction, two researchers independently extracted the required data and cross-checked it. If data was inconsistent, the researchers needed to carefully review the original literature. Missing data should be supplemented by contacting the original author by email, letter, etc. If relevant data was still not available, the literature would be excluded. The following data was required: first author, publication year, study type, general patient characteristics (sample, mean age and proportion of male), type of patients, Whether PCI was performed, treatment dose, platelet function test time and method, definition of HTPR or LTPR, follow-up time, primary outcome (PRU, PRI, AUC, HTPR, LTPR) and secondary outcome (all-cause death, cardiovascular death, myocardial infarction, stroke, target vessel revascularization, stent thrombosis, TIMI minor or minimal bleeding, TIMI major bleeding, bleeding BARC type 1 and bleeding BARC type ≥ 2).

### Risk of Bias Assessment

The literature quality of RCTs was evaluated using the Cochrane handbook (version 6.2) (19), including seven evaluation items: random sequence generation, allocation concealment, blinding of participants and personnel, blinding of outcome assessment, incomplete outcome data, selective reporting, and other biases, each of which was judged to be at low, uncertain or high risk of bias. The literature quality of cohort studies was evaluated using the Newcastle-Ottawa Scale (NOS) (20), including selection of study groups, comparability of groups, and ascertainment of outcomes (cohort studies) or ascertainment of exposure (case-control studies). According to NOS, we could award a cohort study a maximum of four stars in selection, two stars in comparability, and three stars in outcome. The assessment was carried out independently by two researchers, they would cross-check results and resolve a division of opinion through discussion. If it was difficult to reach an agreement, the arbitration would be held in a third researcher.

### Statistical Analysis

We calculated the Cochran's Q-statistic and I^2^ index to estimate heterogeneity between studies. Fixed effects model was adopted if statistical heterogeneity was absent or low (*P* values ≥ 0.1 and I^2^ < 50%). Random effects model was adopted if statistical heterogeneity was significant (*P* values < 0.1 and/or I^2^ ≥ 50%). For continuous data and dichotomous data, the weighted mean difference (WMD) with 95% confidence intervals (CI) and risk ratio (RR) with 95% CI was used, respectively. We conducted the sensitivity analysis by changing the effects model and eliminating literature one by one to establish the robustness of the combined results when statistical heterogeneity was significant. If outcomes were documented in at least ten or more literatures, detection of publication bias was carried out. We adopted the funnel plot, Egger' test, and Begg' test in detection of publication bias (21, 22). Data and figures were analyzed and generated using Review Manager software (version 5.4), STATA (version 15.1), and R software (version 4.0.1). All analyses were two-tailed, with an α of 0.05.

## Results

### Study Selection

**Figure 1:**
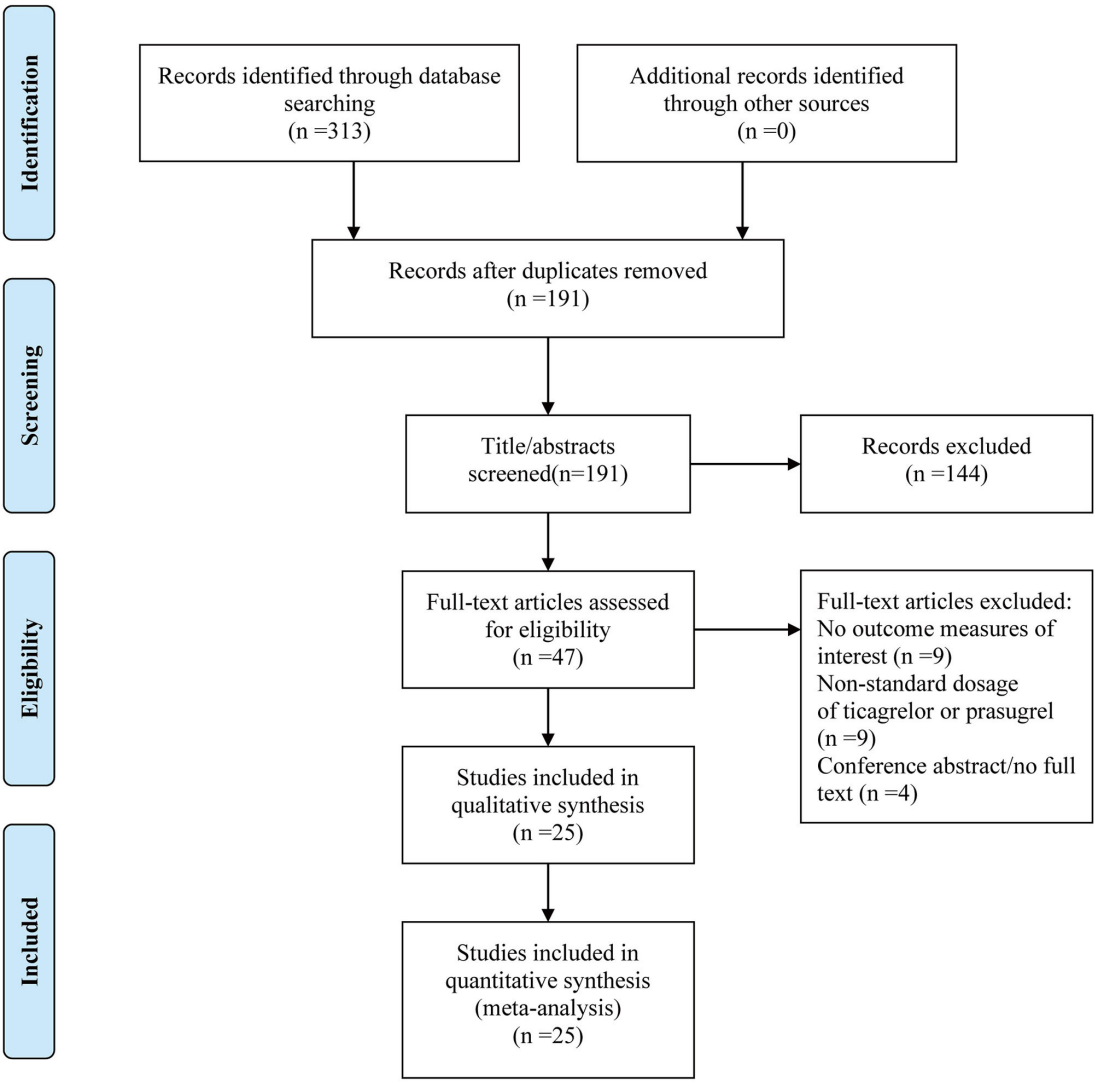
Flow diagram of literature selection and identification.

According to search strategy, we retrieved 313 studies. After removing duplicate studies, 191 studies remained. We screened titles and abstracts and then excluded 144 studies. We conducted a further full-text review of the remaining 47 studies that might meet the eligibility criteria. Finally, 25 (23–47) studies were identified. Detailed screening process is presented in Figure 1.

### Study Characteristics

Among included studies, 11 (25, 26, 29, 30, 33–37, 39, 47) studies were RCTs, 13 (23, 24, 27, 31, 32, 38, 40–46) studies were prospective cohort studies, and one (28) study was retrospective cohort study. The publication dates of 25 studies ranged from 2012 to 2022. The total sample size was 5098 patients, 2600 in ticagrelor group and 2498 in prasugrel group. The patients were all middle-aged and elderly. In terms of Platelet function test method, ten (23, 25, 26, 28–30, 32, 37–39) studies only used VN, six (24, 27, 34–36, 40) studies only used VASP, five studies only used MEA, two (33, 47) studies used VN and VASP, and two (31, 46) studies used VN and MEA. Seven (24, 25, 27, 30, 34, 35, 47) studies only tested platelet function after the treatment of LD. Seventeen (23, 28, 29, 31–33, 36–46) studies only tested platelet function after the treatment of MTD. One (26) study tested platelet function after the treatment of LD and MTD. The characteristic of each selected study is detailed in Table 2.

**Table 2:** Characteristic of selected studies.

**Study**	**Study** **type**	**Sample**	**Mean** **age** **(years)**	**Male** **(%)**	**Type of** **patients**	**PCI**	**Treatment dose**		**PFT** **time**	**PFT** **method**	**Definition**		**Follow** **-up** **time**	**Outcome**	
		**Ticagrelor/** **Prasugrel**	**Ticagrelor/** **Prasugrel**	**Ticagrelor/** **Prasugrel**			**Ticagrelor**	**Prasugrel**			**HTPR**	**LTPR**		**Primary**	**Secondary**
Alexopoulos et al. (26)	RCT	28/27	58.0 ± 12.0/61.0 ± 13.0	86/74	STEMI	Yes	180 mg (LD) then 90 mg bid (MTD)	60 mg (LD) then 10 mg qds (MTD)	1,2,6,24 hours after LD; 5 days after MTD	VN	PRU > 208	NR	5 days	PRU; HTPR	①;④;⑤; ⑦;⑩;⑨;
Alexopoulos et al. (37)	RCT	21/22	61.3 ± 8.1/58.3 ± 8.6	86.4/81.8	STEMI = 43.2%; NSTEMI = 22.7%; UA = 34.1%	Yes	90 mg bid (MTD)	10 mg qd (MTD)	15 days after MTD	VN	NR	NR	15 days	PRU	①;④;⑤; ⑦;⑨;
Alexopoulos et al. (39)	RCT	30/30	65.4 ± 7.7/60.9 ± 8.0	93.3/93.3	ACS	Yes	90 mg bid (MTD)	10 mg qd (MTD)	30 days after MTD	VN	NR	NR	30 days	PRU	①;④;⑤; ⑦;⑧;⑥ ②;③
Alexopoulos et al. (38)	PCS	278/234	60.6 ± 11.8/58.4 ± 10.2	83.5/85.5	ACS	Yes	90 mg bid (MTD)	10 mg qd (MTD)	30 days after MTD	VN	PRU > 208	NR	30 days	PRU; HTPR	②;③;
Alexopoulos et al. (32)	PCS	462/315	60 ± 11 (all)	87 (all)	ACS	Yes	90 mg bid (MTD)	10 mg qd (MTD)	30 days after MTD	VN	PRU > 208	NR	30 days	PRU; HTPR	NR
Deharo et al. (36)	RCT	48/48	60.8 ± 9.8 (all)	81 (all)	ACS	Yes	180 mg (LD) then 90 mg bid (MTD)	60 mg (LD) then 10 mg qd (MTD)	30 days after MTD	VASP	PRI > 50%	PRI ≤ 20%	30 days	PRI; HTPR; LTPR	NR
Dillinger et al. (40)	PCS	119/268	59 (all)	84.5 (all)	STEMI = 28.9%; NSTEMI = 71.1%	NR	90 mg bid (MTD)	10 mg qd (MTD)	Before discharge after MTD	VASP	NR	PRI < 16%	NR	PRI; LTPR	NR
Ferreiro et al. (31)	PCS	446/169	64.5 ± 13.7/57.2 ± 7.1	81.2/82.6	ACS	Yes	180 mg (LD) then 90 mg bid (MTD)	60 mg (LD) then 10 mg qd (MTD)	30 days after MTD	VN; MEA	PRU > 208; AUC > 46.8	PRU < 85; AUC < 18.8	NR	PRU; AUC; HTPR; LTPR	NR
Gager et al. (45)	PCS	260/311	63.0 ± 12.6/57.0 ± 11.2	72/84	STEMI = 64.4%; NSTEMI = 32.6%; UA = 3%	NR	90 mg bid (MTD)	10 mg qd (MTD)	During the treatment with MTD	MEA	AUC > 46	NR	12 months	AUC; HTPR	①;⑥;⑨
Guimarães et al. (30)	RCT	25/25	52.2 ± 8.1/55.5 ± 8.3	72/88	STEMI	Yes	180 mg (LD) then 90 mg bid (MTD)	60 mg (LD) then 10 mg qd (MTD)	2, 6, 24 h after LD	VN	NR	NR	30 days	PRU	②;③
Ibrahim et al. (24)	PCS	22/51	62.6 ± 13.8/56.4 ± 10.6	68/88	ACS	NR	180 mg (LD)	60 mg (LD)	24 h after LD	VASP	NR	NR	NR	PRI	NR
Jin et al. (29)	RCT	40/39	61 ± 9/57 ± 10	85/94.9	ACS	Yes	180 mg (LD) then 90 mg bid (MTD)	60 mg (LD) then 10 mg qd (MTD)	30 days after MTD	VN	NR	NR	9 months	PRU	①;④;⑤; ⑦;⑧;②; ③
Laine et al. (35)	RCT	44/44	57.4 ± 9.8/54.7 ± 8.3	90.9/83.4	STEMI	Yes	180 mg (LD)	60 mg (LD)	6–12 h after LD	VASP	PRI ≥ 50%	PRI ≤ 16%	NR	PRI; HTPR; LTPR	NR
Laine et al. (34)	RCT	50/50	64.8 ± 8.9/62.8 ± 8.2	66/86	STEMI + NSTEMI = 81%; UA = 19%	Yes	180 mg (LD) then 90 mg bid (MTD)	60 mg (LD) then 10 mg qd (MTD)	6–18 h after LD	VASP	PRI ≥ 50%	PRI < 16%	3 ± 2 days	PRI; HTPR; LTPR	①;④;⑤; ⑦
Lee et al. (28)	RCS	24/39	60.1 ± 10.3/56.5 ± 9.5	62.5/92.3	ACS	Yes	180 mg (LD) then 90 mg bid (MTD)	60 mg (LD) then 10 mg qd (MTD)	23.2 ± 7.3 days after MTD	VN	PRU > 208	PRU ≤ 85	23.2 ± 7.3 days	PRU; HTPR; LTPR	①;④;⑤; ⑦;⑧;②; ③
Lhermusier et al. (33)	RCT	10/10	75.0 ± 6.0/64.0 ± 12.0	100/90	ACS	NR	90 mg bid (MTD)	10 mg qd (MTD)	24 ± 4 h after MTD	VN; VASP	NR	NR	NR	PRU; PRI	NR
Motovska et al. (27)	PCS	76/106	65.8 ± 13.3/61.8 ± 11.7	67.1/71.4	ACS	Yes	180 mg (LD)	60 mg (LD)	24 h after LD	VASP	PRI ≥ 50%	NR	NR	PRI; HTPR	NR
Parodi et al. (25)	RCT	25/25	67.0 ± 10.0/67.0 ± 14.0	76/80	STEMI	Yes	180 mg (LD)	60 mg (LD)	2 h after LD	VN	PRU ≥ 240	NR	NR	PRU; HTPR	①;④;⑤; ⑦;⑧;⑩; ⑨
Perl et al. (23)	PCS	52/62	63.2 ± 8.8/57.5 ± 7.6	80.8/79	STEMI	Yes	180 mg (LD) then 90 mg bid (MTD)	60 mg (LD) then 10 mg qd (MTD)	2–4 days after MTD; 30 days after MTD	VN	PRU > 208	NR	30 days	PRU; HTPR	①;④;⑤; ⑦;⑧;⑨
Siller-Matula et al. (44)	PCS	93/107	60.0 ± 13.0/57.0 ± 10.0	71/83	STEMI = 58%; NSTEMI = 42%	Yes	180 mg (LD) then 90 mg bid (MTD)	60 mg (LD) then 10 mg qd (MTD)	During the treatment with MTD	MEA	AUC > 46	AUC < 19	NR	AUC; HTPR; LTPR	NR
Winter et al. (41)	PCS	227/265	63.0 ± 13.0/57.0 ± 11.0	72/86	STEMI = 64%; NSTEMI = 36%	Yes	180 mg (LD) then 90 mg bid (MTD)	60 mg (LD) then 10 mg qd (MTD)	During the treatment with MTD	MEA	AUC > 46	AUC < 19	NR	AUC; HTPR; LTPR	NR
Wadowski et al. (43)	PCS	80/80	59.0 ± 14.1/58.0 ± 11.1	79/81	ACS	Yes	90 mg bid (MTD)	10 mg qd (MTD)	3 days after MTD	MEA	AUC > 46	NR	NR	AUC; HTPR	NR
Wadowski et al. (42)	PCS	80/114	60.0 ± 14.1/57.0 ± 11.1	78/82	ACS	Yes	90 mg bid (MTD)	10 mg qd (MTD)	3 days after MTD	MEA	AUC ≥ 47	NR	NR	AUC; HTPR	NR
Song et al. (46)	PCS	40/38	60.8 ± 8.3/57.7 ± 10.0	85/94.7	STEMI = 62.8%; NSTEMI = 24.4%; UA = 12.8%	Yes	180 mg (LD) then 90 mg bid (MTD)	60 mg (LD) then 10 mg qd (MTD)	30 ± 7 days after MTD	VN; MEA	PRU > 208; AUC > 46	PRU ≤ 85; AUC < 19	90 ± 7 days	PRU; AUC; HTPR; LTPR	NR
Lee et al. (47)	RCT	20/19	55.0 ± 11.0/55.0 ± 10.0	90/89.5	STEMI	Yes	180 mg (LD)	60 mg (LD)	48 h after LD	VN; VASP	PRU > 235; PRI > 50%	PRU < 85; PRI < 16%	NR	PRU; PRI; HTPR; LTPR	NR

① *All-cause death;* ② *Bleeding BARC type 1;* ③ *Bleeding BARC type≥2;* ④ *Cardiovascular death;* ⑤ *Myocardial infarction (MI);* ⑥ *Stent thrombosis(ST);* ⑦ *Stroke;* ⑧ *target vessel revascularization (TVR);* ⑨ *TIMI major bleeding;* ⑩ *TIMI minor or minimal bleeding. CCI, Circulation: Cardiovascular interventions; JACC, Journal of the American College of Cardiology; P, Platelets; TH, Thrombosis and Haemostasis; CJ, Circulation journal: official journal of the Japanese Circulation Society; KJIM, Korean Journal of Internal Medicine; RCT, randomized controlled trial; PCS, prospective cohort study; RCS, retrospective cohort study; ACS, acute coronary syndrome; STEMI, st segment elevation myocardial infarction; NSTEMI, non-st elevation myocardial infarction; UA, unstable angina; PCI, percutaneous coronary intervention; LD, loading dose; MTD, maintenance dose; PFT, platelet function test; VN, verifynow P2Y12 assay; VASP, vasodilator-stimulated phosphoprotein test; MEA, multiple electrode aggregometry test; PRU, P2Y12 reaction unit; PRI, platelet reactivity index; AUC, area under the curve of aggregation tracing; HTPR, high on-treatment platelet reactivity; LTPR, low on-treatment platelet reactivity; NR, not reported*.

### Methodological Quality

**Figure 2:**
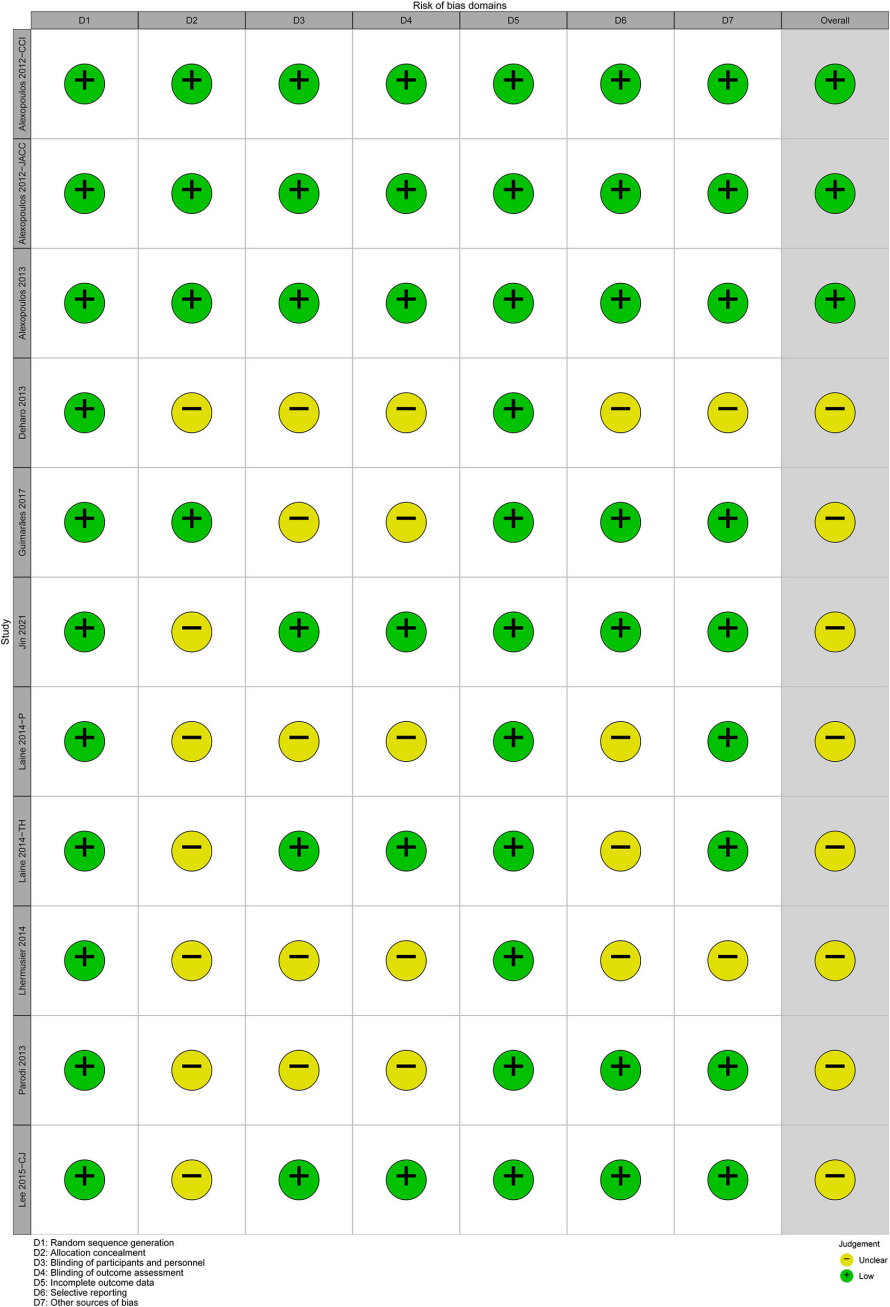
Traffic light plot of risk of bias.

**Figure 3:**
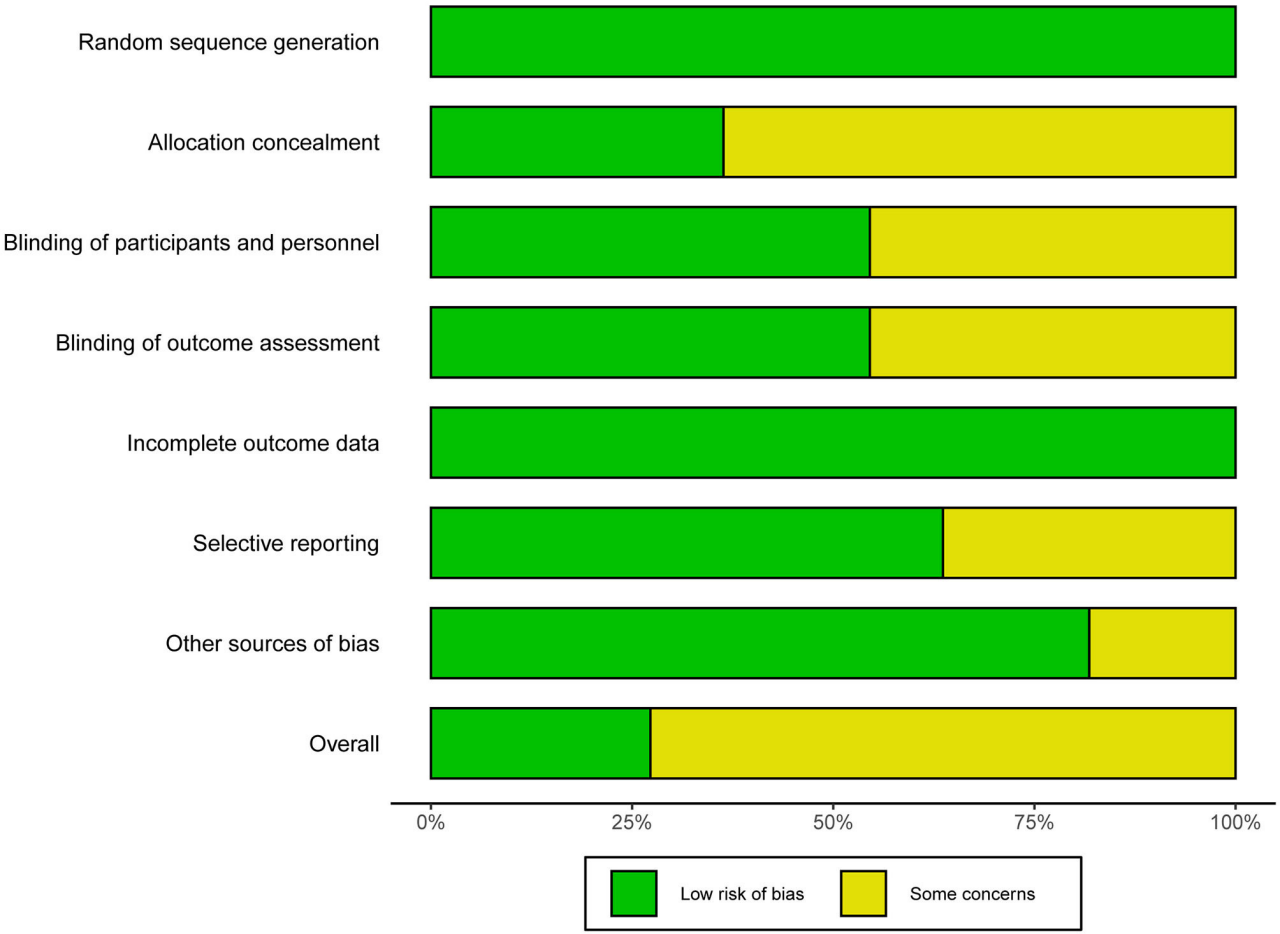
Summary plot of risk of bias.

The overall methodological quality of 11 RCTs was generally high (Figures 2, 3). Three (26, 37, 39) RCTs showed low risk in seven items. Each RCT showed low risk in random sequence generation and incomplete outcome data. Seven (25, 29, 33–36, 47) RCTs showed unclear risk in allocation concealment. Five (25, 30, 33, 35, 36) RCTs showed unclear risk in blinding. Four (33–36) RCTs showed unclear risk in selective reporting. Two (33, 36) RCTs showed unclear risk in other bias.

The overall methodological quality of 14 cohort studies was generally high with the NOS scores ranged from 6 to 9 (Table 3). Six (27, 31, 40, 42–44) studies were rated six stars, three (24, 28, 41) were rated seven stars, four (23, 38, 45, 46) were rated eight stars, and one (32) was rated nine stars.

**Table 3:** Quality assessment of the included cohort studies.

**Study**	**Selection**				**Comparability**	**Outcome**			**Total quality scores**
	**Representativeness of the exposed cohort**	**Selection of the non-exposed cohort**	**Ascertainment of exposure**	**Outcome of interest was not present at start of study**	**Comparability of cohorts on the basis of the design or analysis**	**Assessment of outcome**	**Follow-up long enough for outcomes to occur**	**Adequacy of follow-up of cohorts**	
Alexopoulos et al. (38)	⋆	⋆	⋆	⋆	⋆	⋆	⋆	⋆	8
Alexopoulos et al. (32)	⋆	⋆	⋆	⋆	⋆⋆	⋆	⋆	⋆	9
Dillinger et al. (40)	⋆	⋆	⋆	⋆	⋆	⋆	–	–	6
Ferreiro et al. (31)	⋆	⋆	⋆	⋆	⋆	⋆	–	–	6
Gager et al. (45)	⋆	⋆	⋆	⋆	⋆	⋆	⋆	⋆	8
Ibrahim et al. (24)	⋆	⋆	⋆	⋆	⋆⋆	⋆	–	–	7
Lee et al. (28)	⋆	⋆	⋆	–	⋆	⋆	⋆	⋆	7
Motovska et al. (27)	⋆	⋆	⋆	⋆	⋆	⋆	–	–	6
Perl et al. (23)	⋆	⋆	⋆	⋆	⋆	⋆	⋆	⋆	8
Siller-Matula et al. (44)	⋆	⋆	⋆	⋆	⋆	⋆	–	–	6
Winter et al. (41)	⋆	⋆	⋆	⋆	⋆⋆	⋆	–	–	7
Wadowski et al. (43)	⋆	⋆	⋆	⋆	⋆	⋆	–	–	6
Wadowski et al. (42)	⋆	⋆	⋆	⋆	⋆	⋆	–	–	6
Song et al. (46)	⋆	⋆	⋆	⋆	⋆	⋆	⋆	⋆	8

### PR Meta-Analysis

#### PR After LD

**Figure 4:**
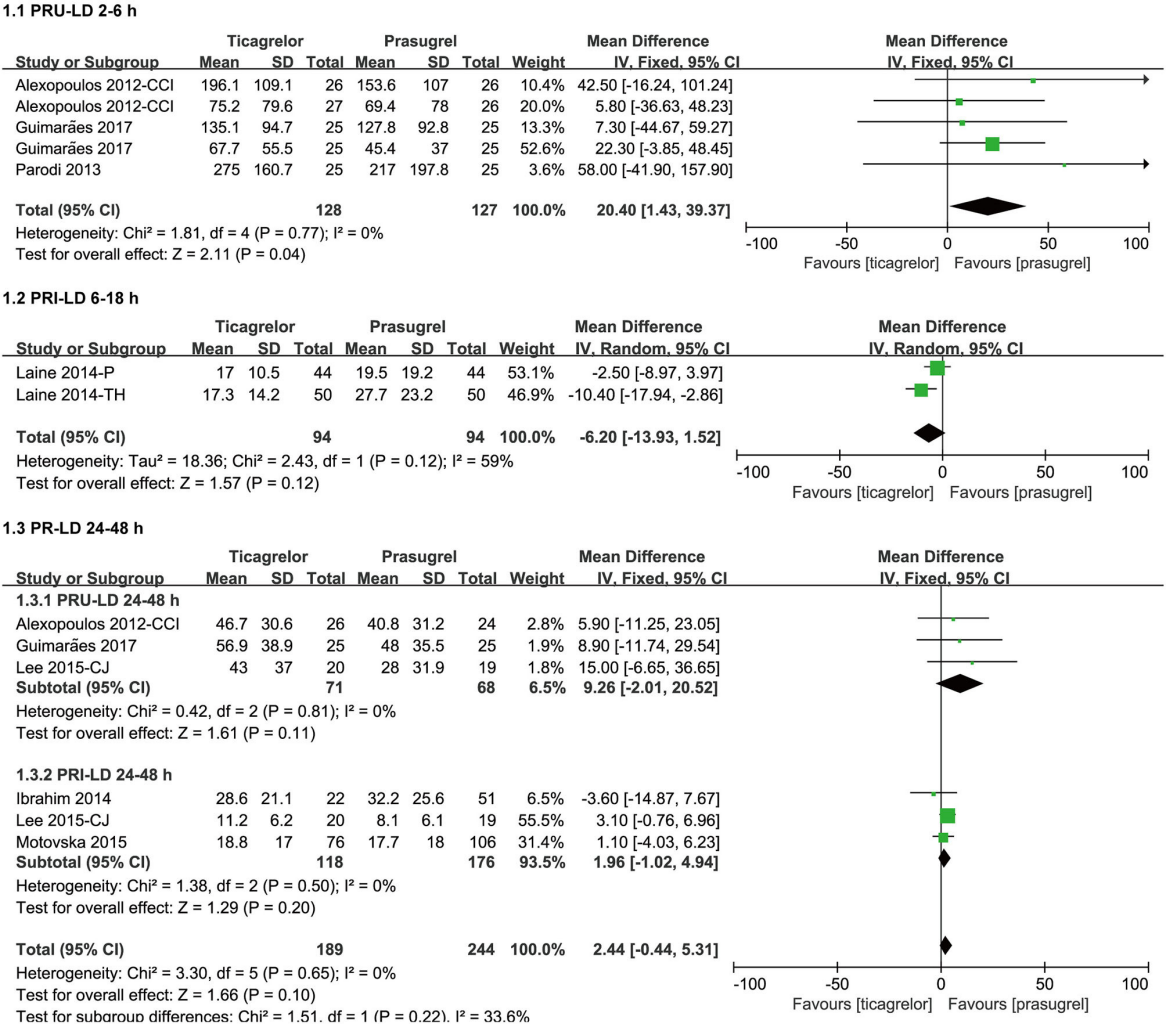
Forest plots of meta-analysis results of PR after LD.

Considering that the LD effect can be separated as early effect and late effect, and the studies containing data of PR after LD used two detection methods (VN and VASP), therefore, we grouped the data of PR after LD according to the detection time (within 2–6 h, within 6–18 h and within 24–48 h) and method. The meta-analysis results of PR after LD were showed in Figure 4.

Three (25, 26, 30) studies which included five sets of data compared PRU after LD within 2–6 h in ticagrelor group and prasugrel group. Ticagrelor group had a significantly higher PRU than prasugrel group [WMD = 20.40 (1.43, 39.37), *P* = 0.04] after LD within 2–6 h. It was worth noting that the *P* value of the overall effect test was 0.04, so though there was no heterogeneity among the five sets of data in the three studies (*P* = 0.77, I^2^ = 0%), sensitivity analysis was still carried out by eliminating studies one by one, and then we found that when eliminating the 6 h data of the study (30), the result substantially changed, the PRU after LD within 2–6 h of the two groups was not significantly different [WMD = 18.28 (−9.29, 45.85), *P* = 0.19] (Supplementary Figure 2).

Two (34, 35) studies compared PRI after LD within 6–18 h in ticagrelor group and prasugrel group. The impact of ticagrelor and prasugrel on PRI after LD within 6–18 h was not significantly different [WMD = −6.20 (−13.93, 1.52), *P* = 0.12, random effects model]. There was moderate heterogeneity among the two studies (*P* = 0.12, I^2^ = 59%). A sensitivity analysis was conducted by changing the effects model, and then found that ticagrelor group had a significantly lower PRI than prasugrel group [WMD = −5.85 (−10.76, −0.94), *P* = 0.02, fixed effects model] after LD within 6–18 h (Supplementary Figure 2), the results of different effect models were inconsistent.

Three (26, 30, 47) studies compared PRU after LD within 24–48 h in ticagrelor group and prasugrel group. The impact of ticagrelor and prasugrel on PRU after LD within 24–48 h was not significantly different [WMD = 9.26 (−2.01, 20.52), *P* = 0.11]. There was no heterogeneity among the three studies (*P* = 0.81, I^2^ = 0%).

Three (24, 27, 47) studies compared PRI after LD within 24–48 h in ticagrelor group and prasugrel group. The impact of ticagrelor and prasugrel on PRI after LD within 24–48 h was not significantly different [WMD = 1.96 (−1.02, 4.94), *P* = 0.20]. There was no heterogeneity among the three studies (*P* = 0.50, I^2^ = 0%).

#### PR After MTD

**Figure 5:**
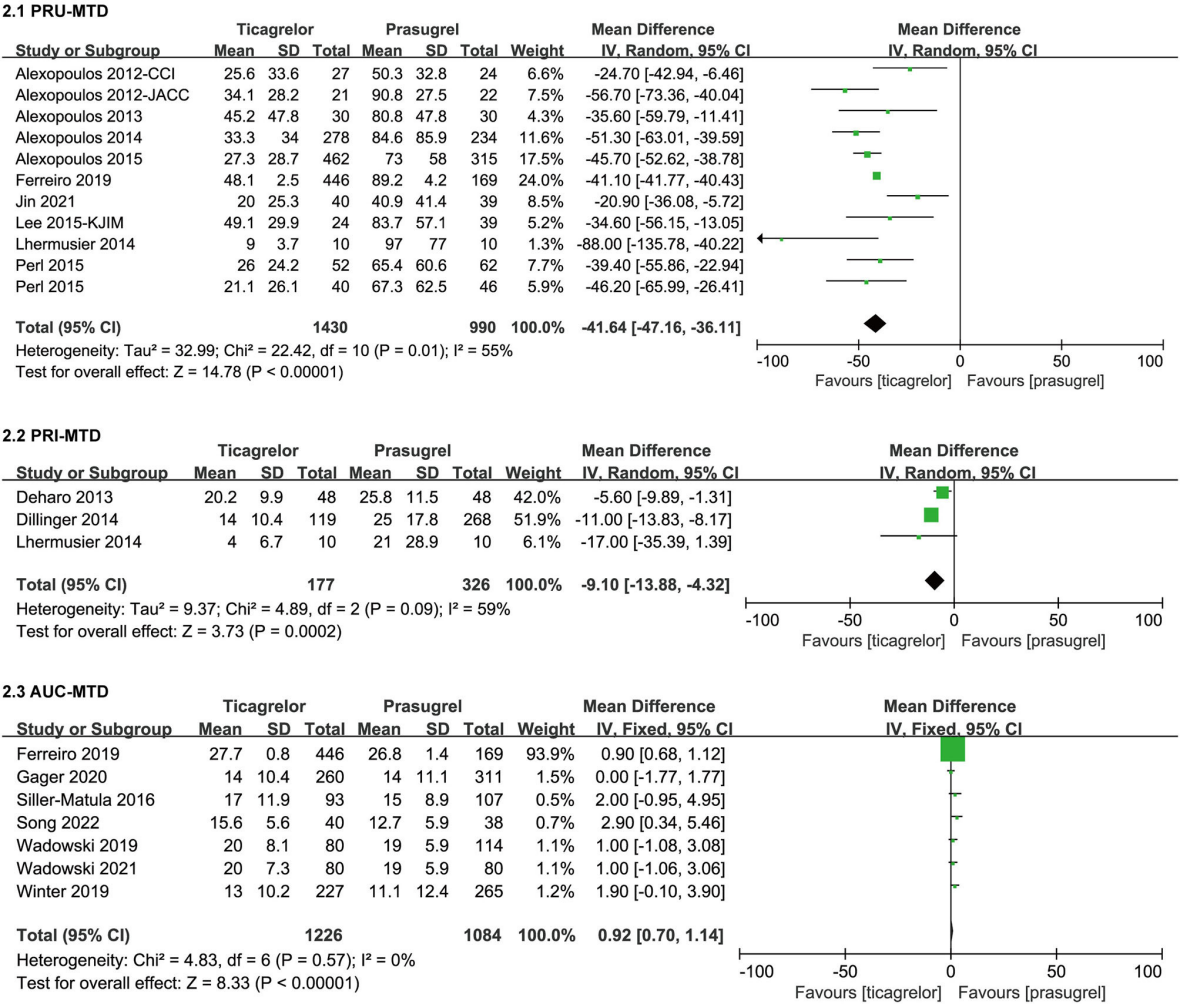
Forest plots of meta-analysis results of PR after MTD.

Studies containing data of PR after MTD used three detection methods (VN, VASP and MEA), therefore, we grouped the data of PR after MTD according to the detection method. The meta-analysis results of PR after MTD were showed in Figure 5.

Song 2022 (46) was the sub-study of Jin et al. (29), therefore, we only included the data of PRU after MTD of Jin et al. for analysis. Eleven (23, 26, 28, 29, 31–33, 37–39, 46) studies which actually included 11 sets of data compared PRU after MTD in ticagrelor group and prasugrel group. After MTD, ticagrelor group had a significantly lower PRU than prasugrel group [WMD = −41.64 (-47.16,−36.11), *P* < 0.00001, random effects model], with moderate heterogeneity among the 11 sets of data (*P* = 0.01, I^2^ = 55%). A sensitivity analysis was conducted by eliminating study one by one and changing the effects model [WMD = −41.14 (−41.81, −40.47), *P* < 0.00001, fixed effects model] (Supplementary Figure 2), the result didn't substantially change.

Three (33, 36, 40) studies compared PRI after MTD in ticagrelor group and prasugrel group. After MTD, ticagrelor group had a significantly lower PRI than prasugrel group [WMD = −9.10 (−13.88, −4.32), *P* = 0.0002, random effects model], with moderate heterogeneity among studies (*P* = 0.09, I^2^ = 59%). A sensitivity analysis was conducted by eliminating study one by one and changing the effects model [WMD = −9.49 (−11.83, −7.14), *P* < 0.00001, fixed effects model] (Supplementary Figure 2), the result didn't substantially change.

Seven (31, 41–46) studies compared AUC after MTD in ticagrelor group and prasugrel group. Ticagrelor group had a slightly higher AUC than prasugrel group [WMD = 0.92 (0.70, 1.14), *P* < 0.00001]. There was no heterogeneity among the seven studies (*P* = 0.57, I^2^ = 0%).

### Meta-Analysis of HTPR and LTPR

#### HTPR After LD

**Figure 6:**
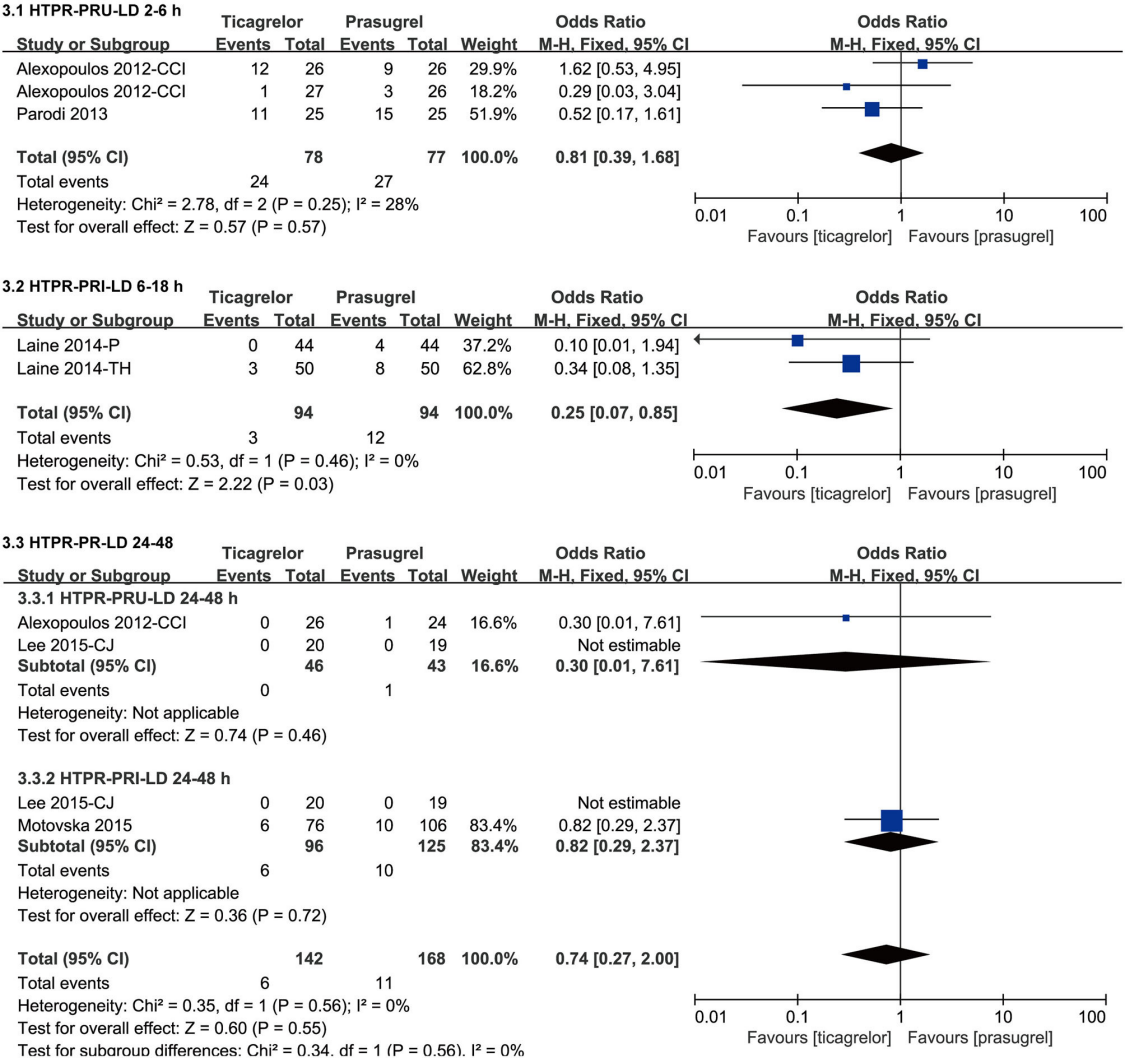
Forest plots of meta-analysis results of HTPR after LD.

Considering that the LD effect can be separated as early effect and late effect, and the studies containing data of HTPR after LD used two detection methods (VN and VASP), therefore, we grouped the data of HTPR after LD according to the detection time (within 2–6 h, within 6–18 h and within 24–48 h) and method. The meta-analysis results of HTPR after LD were showed in Figure 6.

Two (25, 26) studies which included three sets of data compared the incidence of HTPR based on PRU after LD within 2–6 h in ticagrelor group and prasugrel group. According to the VN assay, the incidence of HTPR after LD within 2–6 h of the two groups was not significantly different [RR = 0.81 (0.39, 1.68), *P* = 0.57], with relatively lower heterogeneity among the three sets of data in the two studies (*P* = 0.25, I^2^ = 28%).

Two (34, 35) studies compared the incidence of HTPR based on PRI after LD within 6–18 h in ticagrelor group and prasugrel group. According to the VASP test, ticagrelor group had a significantly lower incidence of HTPR after LD within 6–18 h than prasugrel group [RR = 0.25 (0.07, 0.85), *P* = 0.03, fixed effects model]. It was worth noting that the results of PRI after LD within 6–18 were inconsistent according to different effect models, so though there was no heterogeneity among the two studies (*P* = 0.46, I^2^ = 0%), sensitivity analysis was still carried out by changing the effects model [RR = 0.27 (0.08, 0.95), *P* = 0.04, random effects model] (Supplementary Figure 2), the result didn't substantially change.

Two (26, 47) studies compared the incidence of HTPR based on PRU after LD within 24–48 h in ticagrelor group and prasugrel group. According to the VN assay, the incidence of HTPR after LD within 24–48 h of the two groups was not significantly different [RR = 0.30 (0.01, 7.61), *P* = 0.46].

Two (27, 47) studies compared the incidence of HTPR based on PRI after LD within 24-48 hours in ticagrelor group and prasugrel group. According to the VASP test, the incidence of HTPR after LD within 24–48 h of the two groups was not significantly different [RR = 0.82 (0.29, 2.37), *P* = 0.72].

#### HTPR After MTD

**Figure 7:**
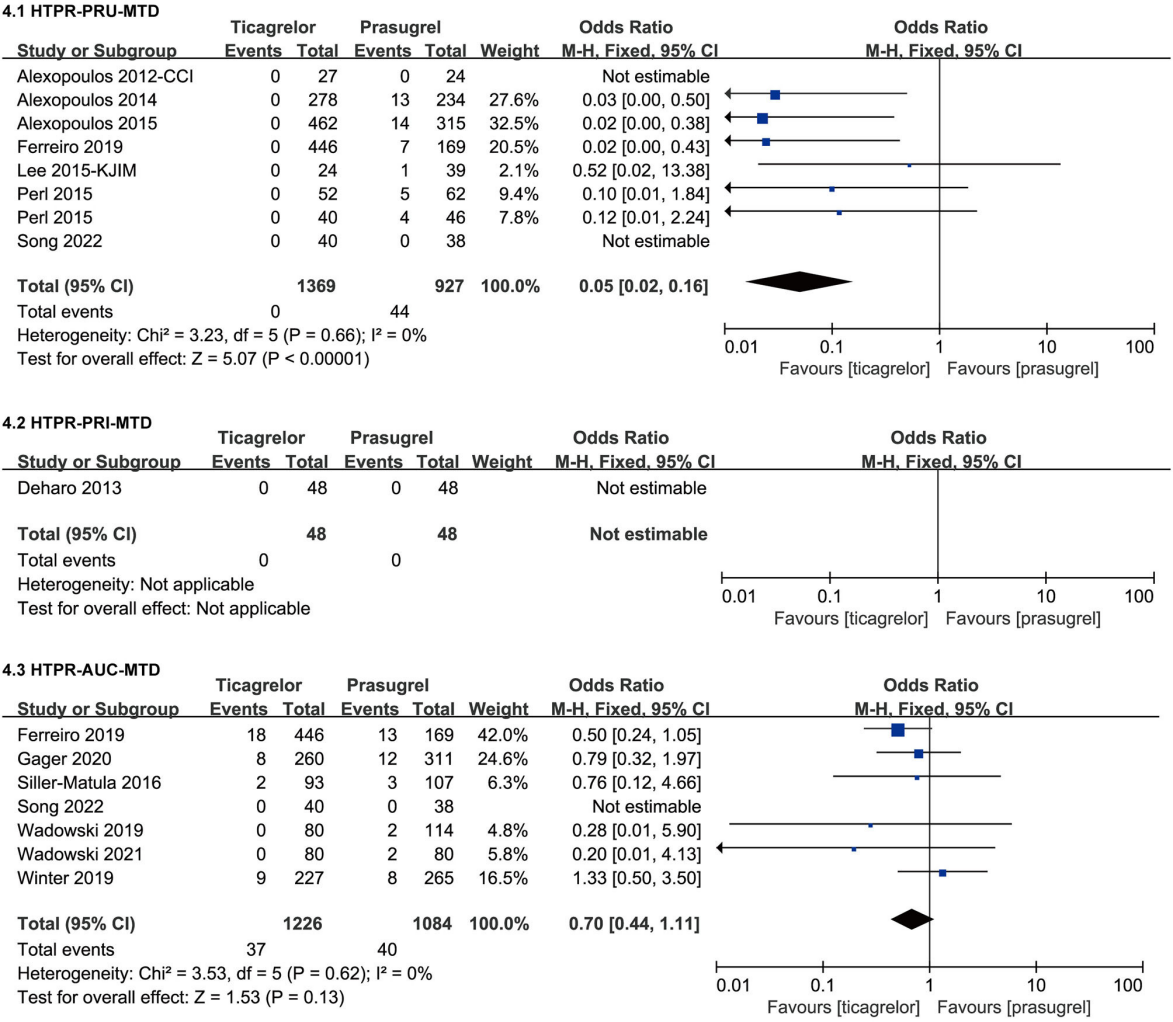
Forest plots of meta-analysis results of HTPR after MTD.

Studies containing data of HTPR after MTD used three detection methods (VN, VASP and MEA), therefore, we grouped the data of HTPR after MTD according to the detection method. The meta-analysis results of HTPR after MTD were showed in Figure 7.

Seven (23, 26, 28, 31, 32, 38, 46) studies which included eight sets of data compared the incidence of HTPR based on PRU after MTD in ticagrelor group and prasugrel group. According to the VN assay, ticagrelor group had a significantly lower incidence of HTPR after MTD than prasugrel group [RR = 0.05 (0.02, 0.16), *P* < 0.00001]. There was no heterogeneity among the eight sets of data in the seven studies (*P* = 0.66, I^2^ = 0%).

Only one (36) study compared the incidence of HTPR based on PRI after MTD in ticagrelor group and prasugrel group. According to VASP test, no HTPR occurred in the two groups after 30 days of MTD treatment.

Seven (31, 41–46) studies compared the incidence of HTPR based on AUC after MTD in ticagrelor group and prasugrel group. According to the MEA test, the incidence of HTPR after MTD of the two groups was not significantly different [RR = 0.70 (0.44, 1.11), *P* = 0.13]. There was no heterogeneity among the seven studies (*P* = 0.62, I^2^ = 0%).

#### LTPR After LD

**Figure 8:**
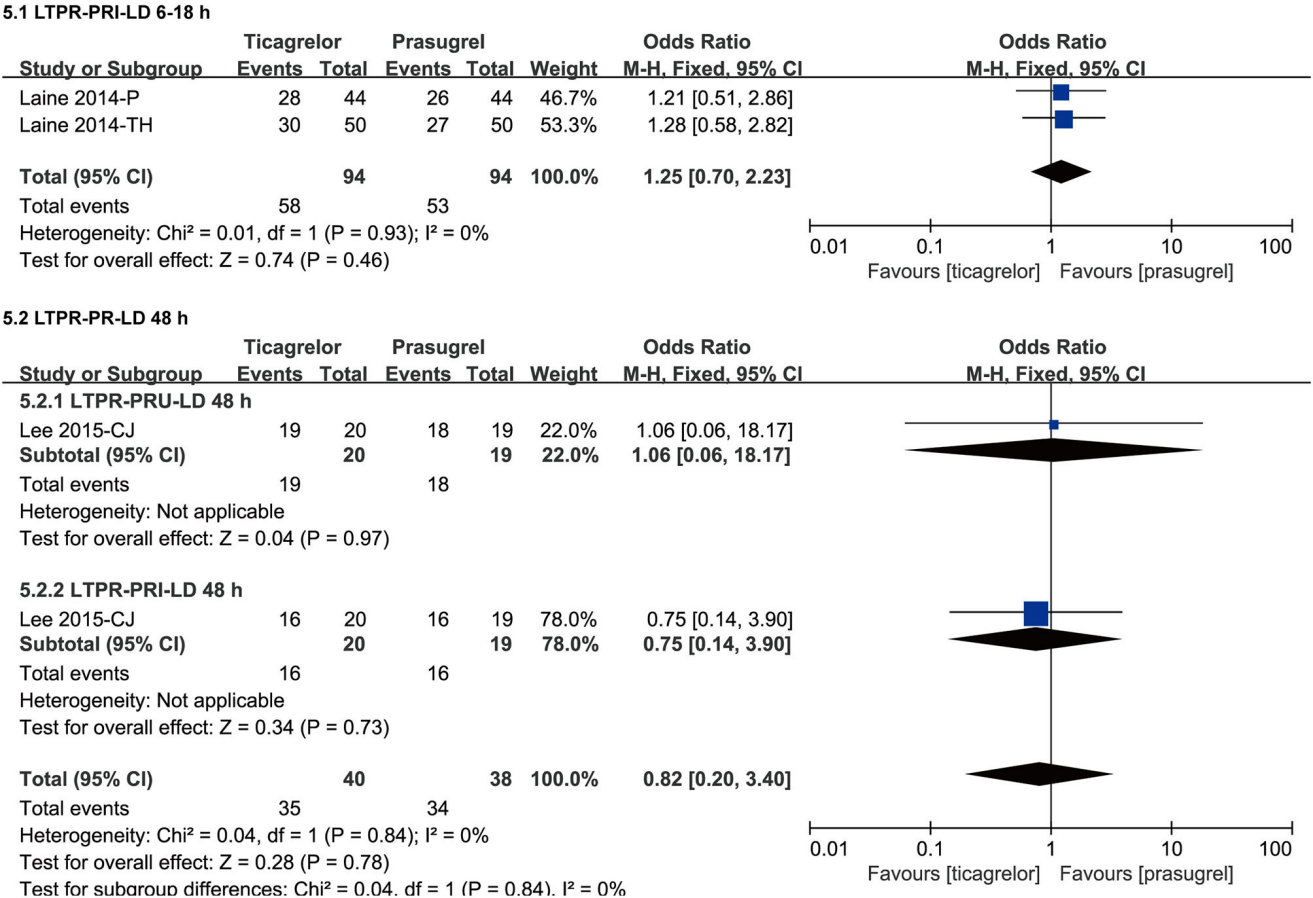
Forest plots of meta-analysis results of LTPR after LD.

Considering that the LD effect can be separated as early effect and late effect, and the studies containing data of LTPR after LD used two detection methods (VN and VASP), therefore, we grouped the data of LTPR after LD according to the detection time (within 6–18 h and 48 h) and method. The meta-analysis results of LTPR after LD were showed in Figure 8.

Two (34, 35) studies compared the incidence of LTPR based on PRI after LD within 6–18 h in ticagrelor group and prasugrel group. According to the VASP test, the incidence of LTPR after LD within 6–18 h of the two groups was not significantly different [RR = 1.25 (0.70, 2.23), *P* = 0.46]. There was no heterogeneity among the two studies (*P* = 0.93, I^2^ = 0%).

One (47) study compared the incidence of LTPR based on both PRU and PRI at 48 h after LD in ticagrelor group and prasugrel group. According to the VN assay and VASP test, the incidence of LTPR at 48 h after LD of the two groups was both not significantly different [PRU, RR = 1.06 (0.06, 18.17), *P* = 0.97; PRI, RR = 0.75 (0.14, 3.90), *P* = 0.73]. There was no heterogeneity among the results based on the two test methods (*P* = 0.84, I^2^ = 0%).

#### LTPR After MTD

**Figure 9:**
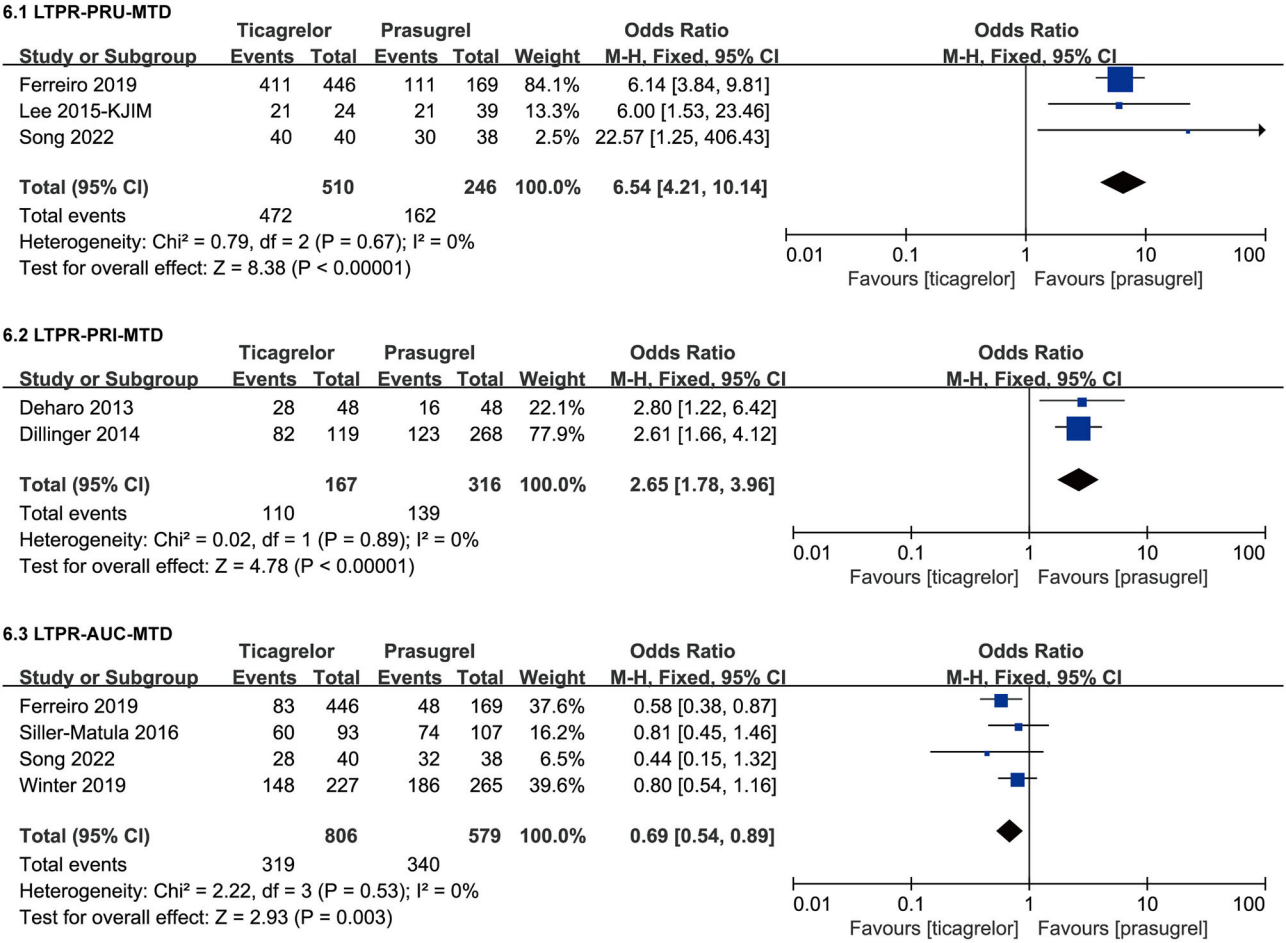
Forest plots of meta-analysis results of LTPR after MTD.

Studies containing data of LTPR after MTD used three detection methods (VN, VASP and MEA), therefore, we grouped the data of LTPR after MTD according to the detection method. The meta-analysis results of LTPR after MTD were showed in Figure 9.

Three (28, 31, 46) studies compared the incidence of LTPR based on PRU after MTD in ticagrelor group and prasugrel group. According to the VN assay, ticagrelor group had a significantly higher incidence of LTPR after MTD than prasugrel group [RR = 6.54 (4.21, 10.14), *P* < 0.00001]. There was no heterogeneity among the three studies (*P* = 0.67, I^2^ = 0%).

Two (36, 40) studies compared the incidence of LTPR based on PRI after MTD in ticagrelor group and prasugrel group. According to the VASP test, ticagrelor group had a significantly higher incidence of LTPR after MTD than prasugrel group [RR = 2.65 (1.78, 3.96), *P* < 0.00001]. There was no heterogeneity among the two studies (*P* = 0.89, I^2^ = 0%).

Four (31, 41, 44, 46) studies compared the incidence of LTPR based on AUC after MTD in ticagrelor group and prasugrel group. According to the MEA test, ticagrelor group had a slightly lower incidence of LTPR after MTD than prasugrel group [RR = 0.69 (0.54, 0.89), *P* = 0.003]. It was worth noting that the result based on AUC was opposite to those based on PRU and PRI, so though there was no heterogeneity among the four studies (*P* = 0.53, I^2^ = 0%), sensitivity analysis was still carried out by eliminating studies one by one, and then we found that when eliminating the study (31), the result substantially changed, the incidence of LTPR based on AUC after MTD of the two groups was not significantly different [RR = 0.76 (0.56, 1.03), *P* = 0.08] (Supplementary Figure 2).

### Meta-Analysis of Clinical Outcomes

The meta-analysis results of ten clinical outcomes were shown in Supplementary Figure 1 and Table 4.

**Table 4:** Meta-analysis results of clinical outcomes.

**Clinical outcome**	**Number of studies**	**Effect estimate**	**Heterogeneity**	**Test for overall effect**
All-cause death	9	0.95 [0.54, 1.66]	*P =* 0.35; I^2^ = 10%	Z = 0.18; *P =* 0.86
Cardiovascular death	8	0.62 [0.16, 2.40]	*P =* 0.21; I^2^ = 37%	Z = 0.69; *P =* 0.49
Myocardial infarction	8	0.70 [0.14, 3.63]	*P =* 0.54; I^2^ = 0%	Z = 0.42; *P =* 0.67
Stroke	8	3.00 [0.12, 76.91]	Not applicable	Z = 0.66; *P =* 0.51
Target vessel revascularization	4	3.00 [0.12, 75.90]	Not applicable	Z = 0.67; *P =* 0.51
Stent thrombosis	3	1.11 [0.19, 6.41]	*P =* 0.33; I^2^ = 0%	Z = 0.12; *P =* 0.90
**TIMI minor or minimal bleeding**	2	6.32 [1.08, 36.89]	*P =* 0.43; I^2^ = 0%	Z = 2.05; ***P =*** **0.04**
TIMI major bleeding	4	1.20 [0.17, 8.56]	Not applicable	Z = 0.18; *P =* 0.86
**Bleeding BARC type 1**	5	1.44 [1.03, 2.02]	*P =* 0.79; I^2^ = 0%	Z = 2.14; ***P =*** **0.03**
Bleeding BARC type ≥ 2	5	0.86 [0.32, 2.32]	*P =* 0.45; I^2^ = 0%	Z = 0.29; *P =* 0.77

*Characters in bold are statistically significant. TIMI, Thrombolysis in Myocardial Infarction; BARC, Bleeding Academic Research Consortium*.

The incidence of the following eight clinical outcomes was not statistically different between ticagrelor and prasugrel [all-cause death, *P* = 0.86; cardiovascular death, *P* = 0.49; myocardial infarction, *P* = 0.67; stroke, *P* = 0.51; target vessel revascularization, *P* = 0.51; stent thrombosis, *P* = 0.90; TIMI major bleeding, *P* = 0.86; bleeding BARC type ≥ 2, *P* = 0.77].

Ticagrelor had a significantly higher incidence of the following two clinical outcomes than prasugrel [TIMI minor or minimal bleeding, RR = 6.32 (1.08, 36.89), *P* = 0.04; bleeding BARC type 1, RR = 1.44 (1.03, 2.02), *P* = 0.03]. The sensitivity analysis was performed by changing the effects model, the result of bleeding BARC type 1 didn't substantially change, however, the result of TIMI minor or minimal bleeding was not robust, the incidence of TIMI minor or minimal bleeding between the two drugs was not significantly different after changing to random effects model [RR = 5.5 (0.88, 34.24), *P* = 0.07] (Supplementary Figure 2).

### Publication Bias

Eleven (23, 26, 28, 29, 31–33, 37–39, 46) studies which included 11 sets of data compared PRU after MTD in ticagrelor group and prasugrel group, so we performed a publication bias to detect the presence of small sample effects, neither funnel plot nor statistical tests found evidence of publication bias (Supplementary Figure 3, Egger's test, *P* = 0.758; Begger's test, *P* = 0.533). There were insufficient studies related to other outcomes, so we could not conduct publication bias test for other outcomes.

## Discussion

PR is an important indicator to evaluate the pharmacodynamic effect of antiplatelet drugs. Results of PR after LD suggested that after LD, the impact of prasugrel and ticagrelor on PRU and PRI within 24–48 h was no significantly different, however the results of the impact on PRU within 2–6 h and on PRI within 6–18 h (only two studies were included) substantially changed after the sensitivity analysis by eliminating literature one by one and changing effects model, which means that the results of 2–6 h and 6–18 h after LD are not robust, more studies are needed. Based on the current data alone, after LD within 24–48 h, there was no difference in the impact of prasugrel and ticagrelor on PR, while within 2–6 h and 6–18 h, we cannot jump to conclusions. Results of PR after MTD suggested that after MTD, the PRU and PRI of ticagrelor group were both significantly lower than those of prasugrel group, however, the AUC of ticagrelor group were slightly higher than that of prasugrel group, the results of VN assay were consistent with that of VASP test, and not with the MEA test. According to VN assay and VASP tests, after MTD, ticagrelor had stronger platelet inhibition than prasugrel, which may be related to different pharmacokinetic and pharmacodynamic properties of the two drugs: prasugrel is a prodrug that irreversibly antagonizes the P2Y12 receptor by conversion to an active metabolite, while ticagrelor does not require metabolic activation to exert activity, binding to P2Y12 receptors in a reversible manner (1), in addition, ticagrelor increases the concentration of plasma adenosine in patients with ACS by inhibiting red blood cell uptake of adenosine, thereby activating A2 adenosine receptors on platelets, increasing intracellular cAMP levels, and inducing VASP phosphorylation by cAMP-dependent protein kinase (48), and the extra adenosine effect of ticagrelor compared to prasugrel may have overestimated the level of PR inhibition assessed by VASP test (40). Research had shown that the results of different platelet function assays differ substantially (49), our study confirmed this once again, there are differences in the assessment of PR between the MEA test and the other two detention methods, which may be related to the sensitivity of the MEA test itself, which suggest that we need to conduct more specialized studies on the consistency of multiple platelet function assays and the correlation between these methods, which may be helpful for personalized antiplatelet therapy guided by PR. On the basis of current results, we can reach the following conclusion, according to VN assay and VASP tests, after LD within 24–48 h, there was no significant difference in platelet inhibition between ticagrelor and prasugrel; after MTD, ticagrelor had stronger platelet inhibition than prasugrel; there are differences in the assessment of PR between the MEA test and the other two detention methods.

Results of HTPR after LD suggested that after LD, the incidence of HTPR in prasugrel group and ticagrelor group within 2–6 h according to VN assay as well as within 24–48 h according to VN assay and VASP test was no significantly different, however within 6–18 h according to VASP test, the incidence of HTPR in prasugrel group was significantly higher than that in ticagrelor group. Results of HTPR after MTD suggested that after MTD, the incidence of HTPR in prasugrel group was significantly higher than that in ticagrelor group according to VN assay, only one study compared the incidence of HTPR after MTD measured by VASP test in the two groups and no HTPR occurred in either group, and according to MEA test, the incidence of HTPR after MTD in prasugrel group and ticagrelor group was no significantly different, which was inconsistent with the results of the VN assay. Some studies have indicated that rate of HTPR may be influenced by different definition and assessment methods (50, 51), this may account for the inconsistency. HTPR had shown to be have a link with increased risk of thrombotic/ischemic events (9). Therefore, based on the above results, we speculated that compared to ticagrelor, prasugrel might have a higher thrombotic/ischemic risk, however, actual clinical results were not the same as we speculated. Recently, Schüpke et al. randomly assigned patients with ACS in a multicenter, randomized, open-label trial and for whom invasive evaluation was planned to receive either ticagrelor or prasugrel (52). In the ISAR-REACT 5 experiment, the incidence of stroke, myocardial infarction, or death in prasugrel group were significantly lower than that in ticagrelor group (52), while according to our meta-analysis results of clinical outcomes, the incidence of thrombotic/ischemic events such as myocardial infarction, stroke, target vessel revascularization, stent thrombosis in two groups was no significant difference. Our included studies predominantly compared PR, studies that met our eligible criteria and reported clinical outcomes were mostly followed up for one month, only two (29, 45) studies followed up for 9 month and 1 year. However, the ISAR-REACT 5 experiment was followed up for 1 year, which may be the reason for the inconsistency with us. Of course, neither our meta-analysis nor the ISAR-REACT 5 experiment found that prasugrel had a higher risk of thrombotic/ischemic than ticagrelor. In summary, although the incidence of HTPR in prasugrel group might be higher than that in the ticagrelor group, comparing the incidence of HTPR between the two drugs alone does not lead to a conclusion of which drug causes a higher risk of thrombosis/ischemia.

Results of LTPR after LD suggested that after LD, the incidence of LTPR in prasugrel group and ticagrelor group was no significantly different within 6–18 h according to VASP test as well as 48 h according to VN assay and VASP test. Results of LTPR after MTD suggested that after MTD, the incidence of LTPR in ticagrelor group was significantly higher than prasugrel group according to VN assay and VASP test, while the result according to MEA assay was not robust, more studies are needed. LTPR had shown to be associated with a higher bleeding risk (53–55). From our findings, it can be speculated that compared to prasugrel, ticagrelor might have a higher bleeding risk. In the ISAR-REACT 5 experiment, major bleeding (BARC type 3 through 5) was observed in 5.4% of patients in the ticagrelor group and 4.8% of patients in the prasugrel group, while there was no significant difference in the incidence of major bleeding between the two groups (52). Our meta-analysis results of clinical outcomes reached a similar conclusion, there was no significant difference of the incidence of TIMI major bleeding, bleeding BARC type ≥ 2 in the two drugs. Besides, we also found that the incidence of bleeding BARC type 1 in ticagrelor group was significantly higher than prasugrel group. In summary, there was no significant difference between ticagrelor and prasugrel in the risk of bleeding BARC Type ≥ 2 and TIMI major bleeding, the incidence of bleeding BARC type 1 in ticagrelor group was significantly higher than prasugrel group and a higher incidence of LTPR in ticagrelor than prasugrel might indicate a higher risk of bleeding BARC type 1.

### Strengths and Limitations

There are several strengths in our meta-analysis. First, in the previous meta-analysis of the impact of ticagrelor and prasugrel on PR, in addition to the PR-related outcomes, no clinical outcomes were included for analysis. However, in our meta-analysis, in addition to comparing the impact of the two drugs on PR and further analyzing the risks of HTPR and LTPR, the clinical outcomes were also included in the analysis, and discussed the association between HTPR and LTPR and thrombosis/ ischemic and bleeding event. Second, in the previous meta-analysis, the impact of the two drugs on PR were analyzed in groups based only on the drug doses (LD and MTD) and two detection methods (VA assay and VASP test). However, we are beyond that also included data of MEA test, and grouped the LD effect by time (within 2–6 h, within 6–18 h, within 24–48 h) to conduct a more detailed analysis. Third, compared with previous meta-analysis of the impact of ticagrelor and prasugrel on PR, we conducted a more comprehensive search and included more literatures, as far as we know, this is the first meta-analysis to assess the incidence of LTPR in the two P2Y12 inhibitors based on VN assay and MEA test. Finally, the studies included in our meta-analysis, both RCTs and cohort studies, had a high overall methodological quality.

There are several limitations of our meta-analysis to be noted. First, our meta-analysis only included studies comparing standard doses of prasugrel and ticagrelor, and the studies comparing the half-dose prasugrel and half-dose ticagrelor were not included. Second, the results of PRU within 2–6 h after LD, PRI within 6–18 h after LD, AUC-based LTPR after MTD were not robust, and only one study compared the incidence of HTPR after MTD measured by VASP test, thus, more large-scale randomized controlled studies are needed for further validation in the future.

## Conclusion

Compared with prasugrel, ticagrelor might have a stronger platelet inhibition effect, with a lower incidence of HTPR and a higher incidence of LTPR and bleeding BARC type 1, while there might be no significant difference in the risk of thrombosis/ischemic, bleeding BARC Type ≥ 2 and TIMI major bleeding. A higher incidence of LTPR might indicate a higher risk of bleeding BARC type 1. The results of VN assay were consistent with that of VASP test, and not with the MEA test.

## Data Availability Statement

The original contributions presented in the study are included in the article/Supplementary Material, further inquiries can be directed to the corresponding author/s.

## Author Contributions

LD and YJ devised the paper. LD and JX identified studies, conducted data collection and extraction, and analyzed the data. LD completed the first draft of the paper. KC provided guidelines for this meta-analysis. All authors approved the final version of the manuscript.

## Funding

This research was financially supported by CACMS Innovation Fund (CI2021A00908) and National Natural Science Foundation of China (81373822).

## Conflict of Interest

The authors declare that the research was conducted in the absence of any commercial or financial relationships that could be construed as a potential conflict of interest.

## Publisher's Note

All claims expressed in this article are solely those of the authors and do not necessarily represent those of their affiliated organizations, or those of the publisher, the editors and the reviewers. Any product that may be evaluated in this article, or claim that may be made by its manufacturer, is not guaranteed or endorsed by the publisher.

## Supplementary Material

Supplementary Figure 1 Forest plots of meta-analysis results of clinical outcomes.

Supplementary Figure 2 Sensitivity analysis for PRU-LD 2-6 h, PRI-LD 6-18 h, PRU-MTD, PRI-MTD, HTPR-PRI-LD 6-18 h, LTPR-AUC-MTD, TIMI Minor or Minimal Bleeding, and Bleeding BARC type 1.

Supplementary Figure 3 Funnel plot of comparison of PRU after MTD between ticagrelor group and prasugrel group.

Supplementary Table 1 Embase search strategy.

Supplementary Table 2 Web of science search strategy.

Supplementary Table 3 Cochrane search strategy.
