# Supplementary figures and images for: Impact of Prasugrel and Ticagrelor on Platelet Reactivity in Patients With Acute Coronary Syndrome: A Meta-Analysis
Source: Front Cardiovasc Med. 2022 Jun 9;9:905607. doi: 10.3389/fcvm.2022.905607 (PMC9226562; doi:10.3389/fcvm.2022.905607)

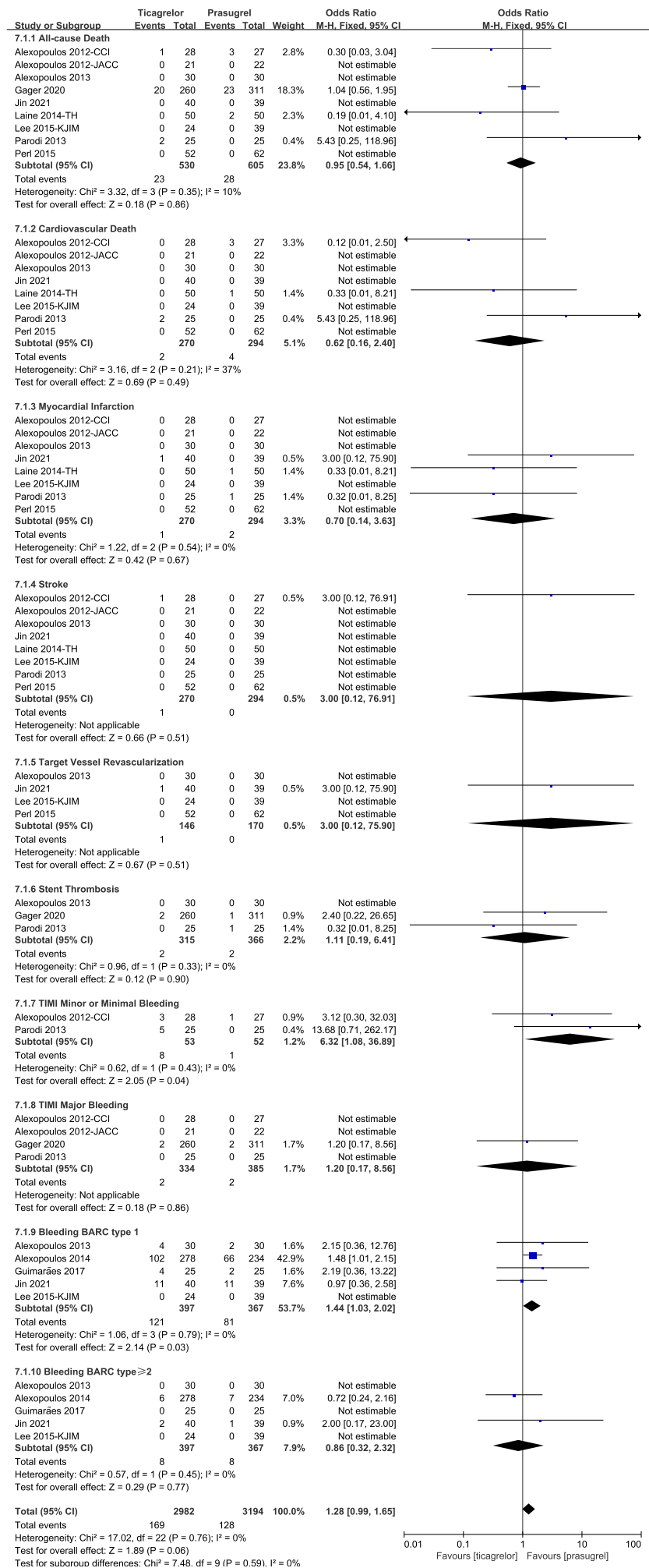

Supplement: Supplementary Figure 1 — Forest plots of meta-analysis results of clinical outcomes. [file Data_Sheet_1.pdf]

Funnel plot with pseudo 95% confidence limits

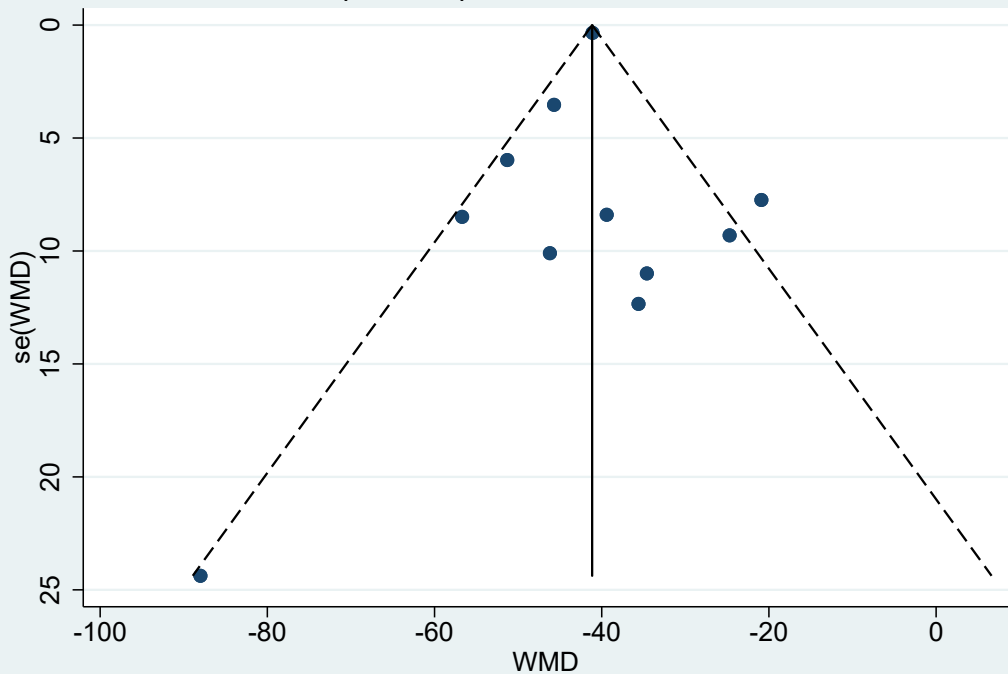

Supplement: Supplementary Figure 3 — Funnel plot of comparison of PRU after MTD between ticagrelor group and prasugrel group. [file Data_Sheet_3.pdf]
