# Supplementary material for: Impact of Prasugrel and Ticagrelor on Platelet Reactivity in Patients With Acute Coronary Syndrome: A Meta-Analysis
Source: Front Cardiovasc Med. 2022 Jun 9;9:905607. doi: 10.3389/fcvm.2022.905607 (PMC9226562; doi:10.3389/fcvm.2022.905607)

Sensitivity analysis for PRU-LD 2-6 h, PRI-LD 6-18 h, PRU-MTD, PRI-MTD, HTPR-PRI-LD 6-18 h, LTPR-AUC-MTD, TIMI Minor or Minimal Bleeding, and Bleeding BARC type 1

1.1 PRU-LD 2-6 h

	Ticagrelor			Prasugrel				Mean Difference
Study or Subgroup	Mean	SD	Total	Mean	SD	Total	Weight	IV, Fixed, 95% CI
Alexopoulos 2012-CCI	196.1	109.1	26	153.6	107	26	22.0%	42.50 [-16.24, 101.24]
Alexopoulos 2012-CCI	75.2	79.6	27	69.4	78	26	42.2%	5.80 [-36.63, 48.23]
Guimarães 2017	135.1	94.7	25	127.8	92.8	25	28.1%	7.30 [-44.67, 59.27]
Guimarães 2017	67.7	55.5	25	45.4	37	25	0.0%	22.30 [-3.85, 48.45]
Parodi 2013	275	160.7	25	217	197.8	25	7.6%	58.00 [-41.90, 157.90]
**Total (95% CI)**			**103**			**102**	**100.0%**	**18.28 [-9.29, 45.85]**

Heterogeneity: Chi² = 1.76, df = 3 (P = 0.62); I² = 0%
Test for overall effect: Z = 1.30 (P = 0.19)

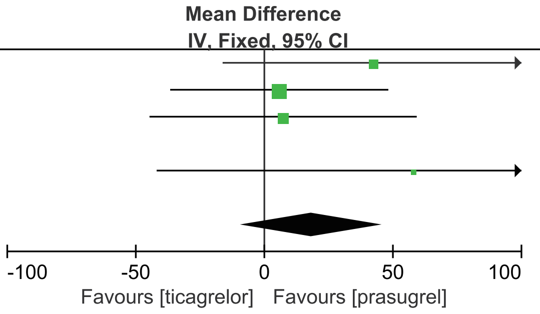

1.2 PRI-LD 6-18 h

	Ticagrelor			Prasugrel				Mean Difference
Study or Subgroup	Mean	SD	Total	Mean	SD	Total	Weight	IV, Fixed, 95% CI
Laine 2014-P	17	10.5	44	19.5	19.2	44	57.6%	-2.50 [-8.97, 3.97]
Laine 2014-TH	17.3	14.2	50	27.7	23.2	50	42.4%	-10.40 [-17.94, -2.86]
**Total (95% CI)**			**94**			**94**	**100.0%**	**-5.85 [-10.76, -0.94]**

Heterogeneity: Chi² = 2.43, df = 1 (P = 0.12); I² = 59%
Test for overall effect: Z = 2.34 (P = 0.02)

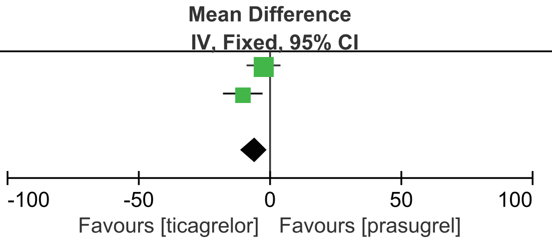

2.1 PRU-MTD

	Ticagrelor			Prasugrel				Mean Difference
Study or Subgroup	Mean	SD	Total	Mean	SD	Total	Weight	IV, Fixed, 95% CI
Alexopoulos 2012-CCI	25.6	33.6	27	50.3	32.8	24	0.1%	-24.70 [-42.94, -6.46]
Alexopoulos 2012-JACC	34.1	28.2	21	90.8	27.5	22	0.2%	-56.70 [-73.36, -40.04]
Alexopoulos 2013	45.2	47.8	30	80.8	47.8	30	0.1%	-35.60 [-59.79, -11.41]
Alexopoulos 2014	33.3	34	278	84.6	85.9	234	0.3%	-51.30 [-63.01, -39.59]
Alexopoulos 2015	27.3	28.7	462	73	58	315	0.9%	-45.70 [-52.62, -38.78]
Ferreiro 2019	48.1	2.5	446	89.2	4.2	169	97.8%	-41.10 [-41.77, -40.43]
Jin 2021	20	25.3	40	40.9	41.4	39	0.2%	-20.90 [-36.08, -5.72]
Lee 2015-KJIM	49.1	29.9	24	83.7	57.1	39	0.1%	-34.60 [-56.15, -13.05]
Lhermusier 2014	9	3.7	10	97	77	10	0.0%	-88.00 [-135.78, -40.22]
Perl 2015	26	24.2	52	65.4	60.6	62	0.2%	-39.40 [-55.86, -22.94]
Perl 2015	21.1	26.1	40	67.3	62.5	46	0.1%	-46.20 [-65.99, -26.41]
**Total (95% CI)**			**1430**			**990**	**100.0%**	**-41.14 [-41.81, -40.47]**

Heterogeneity: Chi² = 22.42, df = 10 (P = 0.01); I² = 55%
Test for overall effect: Z = 120.91 (P < 0.00001)

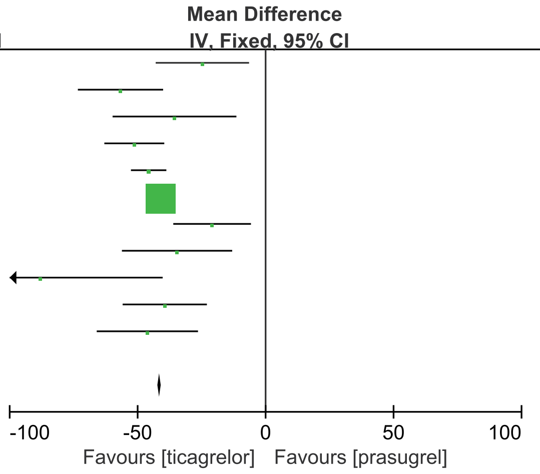

2.2 PRI-MTD

	Ticagrelor			Prasugrel				Mean Difference
Study or Subgroup	Mean	SD	Total	Mean	SD	Total	Weight	IV, Fixed, 95% CI
Deharo 2013	20.2	9.9	48	25.8	11.5	48	29.9%	-5.60 [-9.89, -1.31]
Dillinger 2014	14	10.4	119	25	17.8	268	68.5%	-11.00 [-13.83, -8.17]
Lhermusier 2014	4	6.7	10	21	28.9	10	1.6%	-17.00 [-35.39, 1.39]
**Total (95% CI)**			**177**			**326**	**100.0%**	**-9.49 [-11.83, -7.14]**

Heterogeneity: Chi² = 4.89, df = 2 (P = 0.09); I² = 59%
Test for overall effect: Z = 7.92 (P < 0.00001)

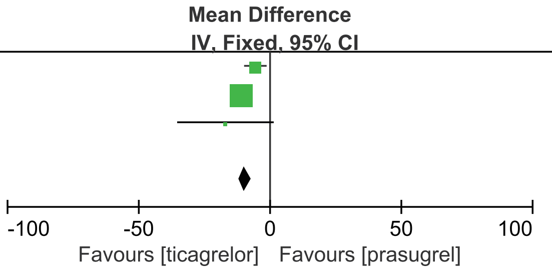

3.2 HTPR-PRI-LD 6-18 h

	Ticagrelor		Prasugrel			Odds Ratio
Study or Subgroup	Events	Total	Events	Total	Weight	M-H, Random, 95% CI
Laine 2014-P	0	44	4	44	18.2%	0.10 [0.01, 1.94]
Laine 2014-TH	3	50	8	50	81.8%	0.34 [0.08, 1.35]
**Total (95% CI)**		**94**		**94**	**100.0%**	**0.27 [0.08, 0.95]**
Total events	3		12			

Heterogeneity: Tau² = 0.00; Chi² = 0.53, df = 1 (P = 0.46); I² = 0%
Test for overall effect: Z = 2.04 (P = 0.04)

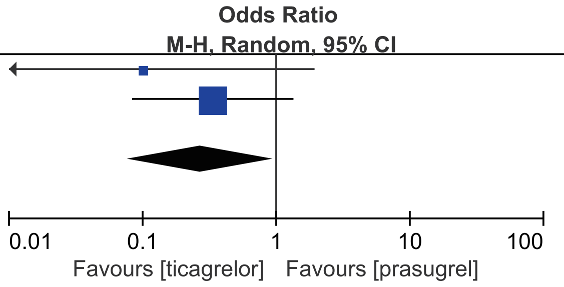

6.3 LTPR-AUC-MTD

	Ticagrelor		Prasugrel			Odds Ratio
Study or Subgroup	Events	Total	Events	Total	Weight	M-H, Fixed, 95% CI
Ferreiro 2019	83	446	48	169	0.0%	0.58 [0.38, 0.87]
Siller-Matula 2016	60	93	74	107	26.0%	0.81 [0.45, 1.46]
Song 2022	28	40	32	38	10.5%	0.44 [0.15, 1.32]
Winter 2019	148	227	186	265	63.5%	0.80 [0.54, 1.16]
**Total (95% CI)**		**360**		**410**	**100.0%**	**0.76 [0.56, 1.03]**
Total events	236		292			

Heterogeneity: Chi² = 1.06, df = 2 (P = 0.59); I² = 0%
Test for overall effect: Z = 1.74 (P = 0.08)

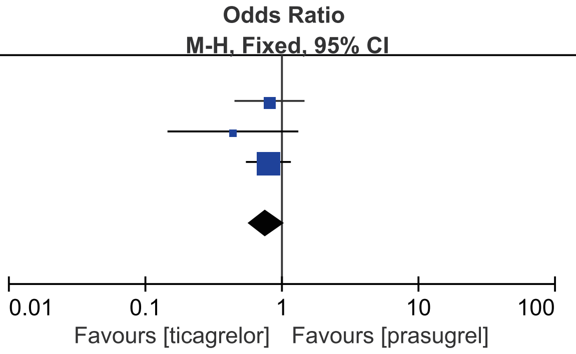

7.1.7 TIMI Minor or Minimal Bleeding

	Ticagrelor		Prasugrel			Odds Ratio
Study or Subgroup	Events	Total	Events	Total	Weight	M-H, Random, 95% CI
Alexopoulos 2012-CCI	3	28	1	27	61.7%	3.12 [0.30, 32.03]
Parodi 2013	5	25	0	25	38.3%	13.68 [0.71, 262.17]
**Total (95% CI)**		**53**		**52**	**100.0%**	**5.50 [0.88, 34.24]**
Total events	8		1			

Heterogeneity: Tau² = 0.00; Chi² = 0.62, df = 1 (P = 0.43); I² = 0%
Test for overall effect: Z = 1.83 (P = 0.07)

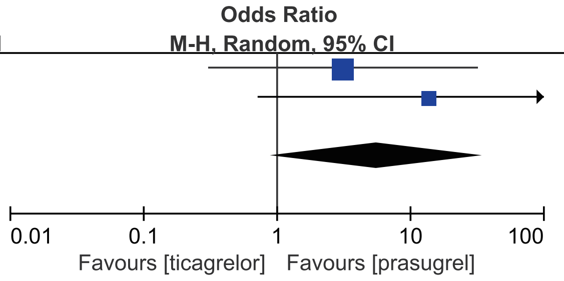

7.1.9 Bleeding BARC type 1

	Ticagrelor		Prasugrel			Odds Ratio
Study or Subgroup	Events	Total	Events	Total	Weight	M-H, Random, 95% CI
Alexopoulos 2013	4	30	2	30	3.6%	2.15 [0.36, 12.76]
Alexopoulos 2014	102	278	66	234	81.1%	1.48 [1.01, 2.15]
Guimarães 2017	4	25	2	25	3.5%	2.19 [0.36, 13.22]
Jin 2021	11	40	11	39	11.8%	0.97 [0.36, 2.58]
Lee 2015-KJIM	0	24	0	39		Not estimable
**Total (95% CI)**		**397**		**367**	**100.0%**	**1.44 [1.03, 2.02]**
Total events	121		81			

Heterogeneity: Tau² = 0.00; Chi² = 1.06, df = 3 (P = 0.79); I² = 0%
Test for overall effect: Z = 2.13 (P = 0.03)

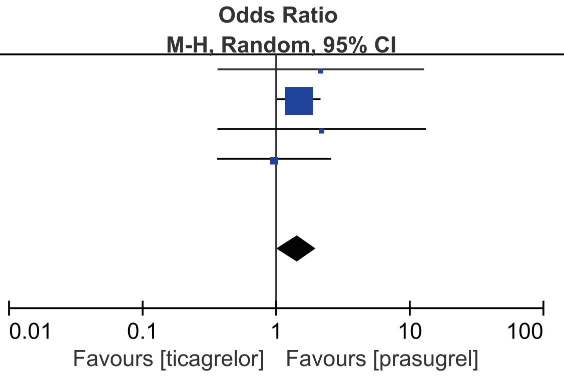

Supplement: Supplementary Figure 2 — Sensitivity analysis for PRU-LD 2-6 h, PRI-LD 6-18 h, PRU-MTD, PRI-MTD, HTPR-PRI-LD 6-18 h, LTPR-AUC-MTD, TIMI Minor or Minimal Bleeding, and Bleeding BARC type 1. [file Data_Sheet_2.pdf]
